# Esketamine ameliorates depression-like behavior in mice via modulation of the NRG1–ErbB4 pathway

**DOI:** 10.3389/fpsyt.2026.1722336

**Published:** 2026-04-07

**Authors:** Hang Yu, Yuqiong Zhu, Jie Wang, Yifan He, Hui Li, Shuxuan Li, Ning Wang, Nuo Chen, Juan Chen, Hongtao Song, Mingjie Zhang, Wenjuan Wang

**Affiliations:** 1 School of Mental Health, Bengbu Medical University, Bengbu, Anhui, China; 2 Linping Hospital of Integrated Traditional Chinese and Western Medicine, Hangzhou, Zhejiang, China; 3 Hefei University of Technology, Hefei, China; 4 The First Affiliated Hospital of Bengbu Medical University, Bengbu, Anhui, China

**Keywords:** chronic social defeat stress (CSDS), depression disorder, ErbB4, esketamine, NRG1

## Abstract

**Background:**

Esketamine has a significant and rapid antidepressant effect. Although studies have shown that Neuregulin 1 (NRG1) and it’s signaling pathway are associated with depression, the possible regulatory relationship of esketamine on the NRG1-ErbB4 pathway is not yet clear.

**Methods:**

To induce depressive-like behavior in mice, a Chronic Social Defeat Stress (CSDS) model was established. Behavioral indicators were then employed to assess depression in these mice, categorized into control, susceptible, and resilient groups. Following intraperitoneal injection of a subanesthetic dose of esketamine, behavioral tests were conducted at 30 minutes and 24 hours post-injection to observe any improvements in depressive-like behavior. Additionally, changes in immunofluorescence and protein expression levels of NRG1-ErbB4 and GAD67 in the prefrontal cortex were evaluated.

**Results:**

Compared with the control group, the CSDS susceptible group mice showed decreases in social interaction ratio in the contact area, sucrose preference ratio, NRG1 immunofluorescence protein expression in the prefrontal cortex and NRG1 expression in tissue homogenate; showed significant increases in immobility time; the expression of NRG1 decreased;no significant change in GAD67 and ErbB4 expression level. in After 30 minutes of intraperitoneal injection of esketamine, the expression of NRG1 in the prefrontal cortex of susceptible mice increased significantly. no significant change in GAD67 and ErbB4 expression level. After 30 minutes and 24 hours of intraperitoneal injection of esketamine, the social interaction ratio of susceptible group improved compared to the control group, and the duration of forced swimming immobility was significantly shortened.

**Conclusion:**

The subanesthetic dose of esketamine may regulate the NRG1-ErbB4 signaling pathway and improve depressive like behavior in mice.

## 1 Background

Major Depressive Disorder (MDD) is a common mental disorder characterized by persistent low mood, diminished interest in daily activities, and loss of pleasure, which may lead to slowed thinking, cognitive impairment, and a variety of physical symptoms, and may even lead to suicidal behavior. A 2023 WHO study revealed that 3.8% of the world’s population struggles with depression, with adults accounting for an alarming 5% of these cases. This statistic underscores the widespread and significant impact of MDD globally. This neuropsychiatric condition is intricate, with its origins stemming from a combination of social stressors, psychological influences, and biological factors (1).

Traditional antidepressants, such as selective serotonin reuptake inhibitors (SSRIs), require prolonged use, have a delayed onset of action, may diminish in effectiveness over time, and can cause withdrawal symptoms upon abrupt cessation (2). In contrast, a single low-dose infusion of ketamine can produce clinically evident mood improvement within hours, prompting investigations into its rapid mechanism (3). Ketamine, a non-specific NMDA receptor antagonist, particularly its enantiomer esketamine, shares a similar mechanism of action and exhibits rapid fat-solubility, with a two-fold affinity compared to racemic ketamine. It swiftly crosses the blood-brain barrier within five minutes of injection, exhibiting prompt and notable antidepressant effects, a short half-life, swift recovery, mild respiratory depression, and minimal psychiatric side effects, garnering significant attention (4, 5). Esktamine has emerged as a research focus due to its rapid-onset effects and unique mechanism of action, which involve synaptic plasticity and neurotrophic signaling (6, 7).

Neuregulins (NRGs) are growth factors structurally similar to epidermal growth factor (EGF) and signal through ErbB tyrosine kinase receptors. NRG1, encoded by one of the largest human genes, performs diverse functions in the central nervous system, producing various isoforms through alternative splicing and promoter usage. Dysregulation of the NRG1 gene is strongly linked to psychiatric disorders like schizophrenia and bipolar disorder. NRG1 expression is observed in multiple brain regions, including the prefrontal cortex (PFC), hippocampus, cerebellum, hypothalamus, and substantia nigra (8). As a crucial signaling molecule on axon surfaces, NRG1 communicates with Schwann cells throughout development, regulating myelin sheath thickness (9). NRG1 plays pivotal roles in the development and function of the central nervous system, such as promoting synaptic formation and plasticity, regulating intercellular signaling, facilitating neuronal migration, and promoting glial cell differentiation (10). Additionally, NRG1 is vital for neuronal proliferation and regeneration.

NRG1 may be implicated in the pathophysiology and treatment of depressive disorders. Increased NRG1 mRNA expression in the serum of MDD patients has been noted (11), and dysregulation of the NRG1 gene rs6994992 correlates with depression severity (12). Decreased NRG1-ErbB signaling is observed in chronic schizophrenic patients with depressive phenotypes (13), along with altered NRG1 and ErbB mRNA levels in the peripheral blood mononuclear cells of depressive disorder patients (14). Genome-wide association studies of antidepressant treatment revealed that NRG1 genetic variations in depressed patients can influence SSRI efficacy (15). In rat models of depression, reduced NRG1 expression in the hippocampus and plasma NRG1 concentrations were observed (16). Hippocampal NRG1 administration enhanced neurogenesis and counteracted depressive behaviors (17). Postpartum depression model rats exhibited decreased NRG1 levels in the cortex, with reduced dendritic spines in the medial prefrontal cortex and nucleus accumbens (18). These findings suggest that NRG1 dysregulation may play a significant role in the pathogenesis of depression in humans and animal models.

ErbB2 to ErbB4 are the primary receptors for NRG1 signaling. ErbB2 lacks ligand-binding domains, while ErbB3 lacks tyrosine kinase activity and functions as a co-receptor for EGFR. Only ErbB4 can bind ligands, become activated, and form tyrosine kinases (19). In the central nervous system, NRG1 signaling predominantly occurs through ErbB4. ErbB4 is a transmembrane protein comprising a tyrosine kinase domain, inositol triphosphate (IP3) binding site, and PDZ domain. NRG1 activates ErbB4, inducing conformational changes in its extracellular domain, binding to other signaling molecules, and regulating cell proliferation and damage repair through gene transcription (20).

As a key component of the emotional pathway, PFC activity changes are crucial for regulating stress-induced symptoms (21), and are often compromised under stress. Chronic stress reduces PFC connectivity with adjacent regions (22) and glial cell proliferation (23). γ- aminobutyric acid (GABA) synthesis primarily occurs through glutamic acid decarboxylation, with GAD67 converting glutamate to GABA, crucial for neuronal excitability and inhibition, playing a vital role in nervous system development and function.

Ketamine, an NMDA receptor antagonist, preferentially binds to NMDA receptors on GABAergic interneurons. These interneurons, characterized by their high firing rates, are more likely to relieve the Mg²^+^ block through activity, thus enabling ketamine to selectively engage with the NMDA receptor channel pores on these neurons. This selective binding inhibits the activity of GABAergic interneurons, leading to disinhibition of pyramidal cells and an enhancement of their activity (24, 25). PV interneurons are crucial targets for NRG1-ErbB4 signaling, with ErbB4 on mouse brain PV interneurons playing a significant role in NRG1-regulated long-term potentiation (26). The release of GABA is regulated by NRG1-ErbB4 signaling, which promotes functional recovery in traumatic brain injury (27). These findings suggest that ketamine may exert rapid antidepressant effects through the NRG1-ErbB4 signaling pathway.

Acute sub-anesthetic ketamine activates prefrontal NRG1–ErbB4 signaling and exerts rapid antidepressant-like effects in stress-naïve rodents (28, 29). Whether the esketamine retains this molecular action after chronic social defeat stress (CSDS), and whether such signaling is obligatory for its sustained reversal of stress-evoked social avoidance and anhedonia, is unknown. To close this gap, we therefore tested the hypothesis that esketamine-induced re-activation of prefrontal NRG1–ErbB4 signaling is both necessary and sufficient for the sustained reversal of CSDS-evoked social avoidance and anhedonia. Aim to delineate a causal link between esketamine-induced NRG1–ErbB4 engagement and its long-lasting antidepressant actions in stress-sensitized brains.

## 2 Materials and methods

### 2.1 Animals

C57BL/6J male mice, aged 6–8 weeks and weighing 18-22g, and CD-1 male mice, aged 6–8 months and weighing at least 24g (retired breeders), were obtained from Henan Skobes Biotechnology Co. The animals were maintained in a standard environment with a temperature range of 18-22 °C, a 12-hour light/dark cycle, and ad libitum access to food and water. Animal experiments were approved by the Institutional Animal Care and Use Committees of Bengbu Medical University and conducted in accordance with the UK Animals (Scientific Procedures) Act, 1986. Ethical approval was granted under number [2023] No. 661.

In the animal experiments, we administered pentobarbital sodium by intraperitoneal injection to carry out euthanasia, ensuring that the animals quickly entered a deep state of anesthesia, eventually experiencing respiratory and cardiac arrest and death, passing away in a painless and fear-free state.

### 2.2 Experimental design

**Figure 1:**
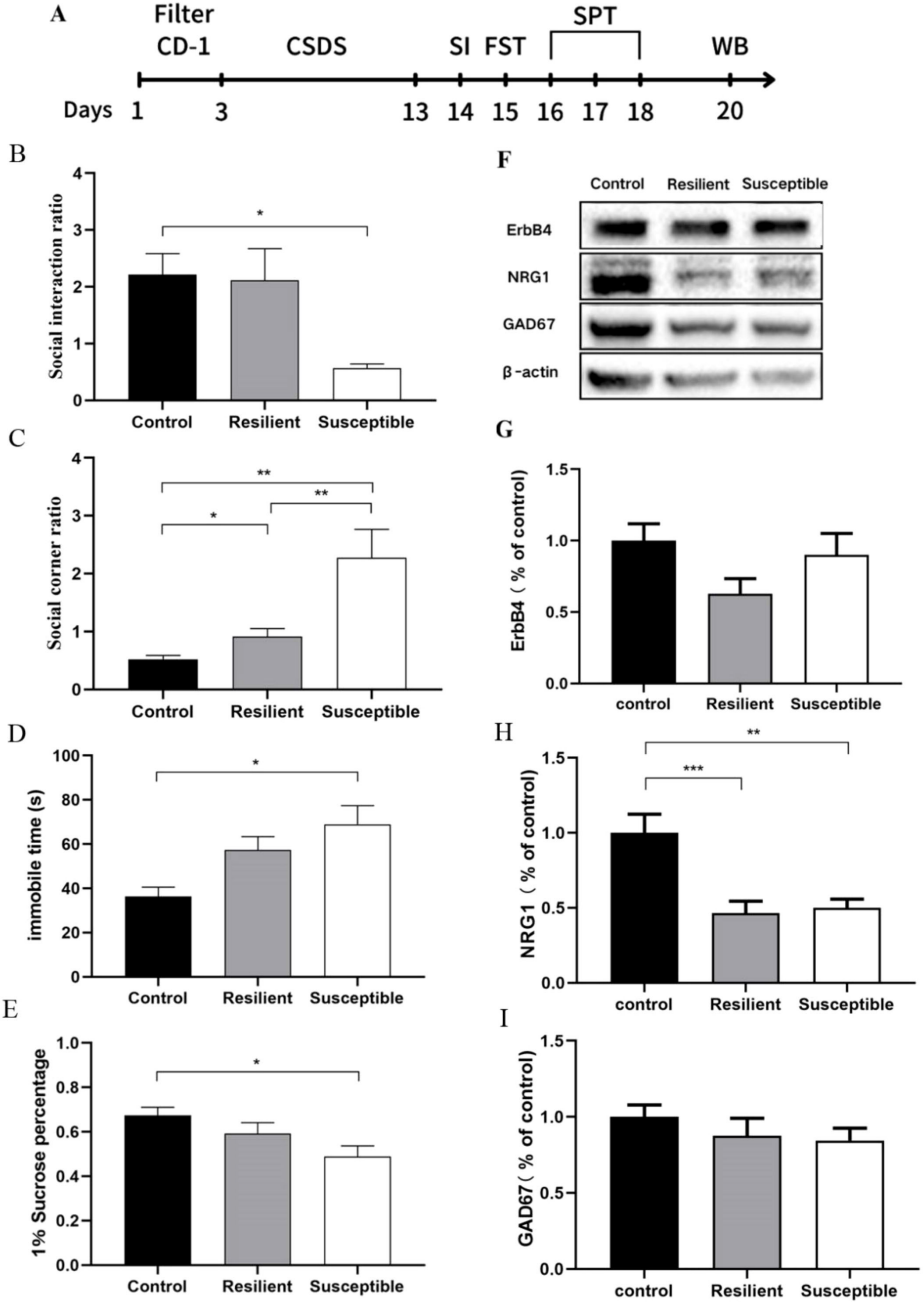
CSDS model process and behavioral experiments and protein changes in PFC of mice after CSDS modeling. **(A)** Experimental flowchart; **(B)** Social interaction ratio is the ratio of the activity time of C57BL/6J mice in the social contact area during the behavioral testing stage, with and without CD-1 mice (n: control = 15, resilient = 13, susceptible = 11); **(C)** The ratio of activity time in the corner area of C57BL/6J mice with and without CD-1 mice (n: control = 15, resilient = 13, susceptible = 11); **(D)** forced swimming experiment (n: control = 15, resilient = 13, susceptible = 11); **(E)** sugar water preference experiment (n: control = 15, resilient = 13, susceptible = 11); **(F)** Changes in PFC protein expression after CSDS modeling in WB results: control group, resilient group and susceptible group from left to right, while from top to bottom are ErbB4, NRG1, GAD67, and β-actin; **(G)** There was no significant difference in the expression level of ErbB4 after modeling with CSDS (n: control = 6, resilient = 10, susceptible = 8); **(H)** After modeling with CSDS, the expression level of NRG1 in PFC of susceptible group mice and resilient group mice decreased (n: control = 7, resilient = 9, susceptible = 9); **(I)** There was no significant difference in the expression level of GAD67 after modeling with CSDS (n: control = 7, resilient = 9, susceptible = 9). All data are expressed as mean ± SEM, *P < 0.05, **P < 0.01, ***P < 0.001, one-way ANOVA.

A CSDS model was utilized to induce depressive-like behavior in mice, with social interaction indicators serving as the basis for evaluating depression in these mice. The modeled mice were categorized into control, susceptible, and resilient groups. The drug group was further divided into two subgroups (saline and 10mg/kg esketamine) across the three aforementioned groups. Determined the necessary amount of esketamine for each mouse based on its weight, and dissolved this quantity in saline to reach the desired concentration. Used an electronic scale to precisely measure each mouse’s weight. Subsequently, calculated the individual dose for each mouse and administered it via intraperitoneal injection using sterile methods. Following drug administration the mice underwent behavioral testing, as illustrated in **Figure 1A**.

### 2.3 Tissue preparation

Brain tissues were meticulously prepared for staining and blotting procedures. The brains were cryoprotected in 30% sucrose, then cut into 30 µm coronal sections on a freezing microtome. Finally, they were stored in cryoprotectant at –20 °C, ready for immunofluorescence. The antibodies used were Rabbit anti-NRG1 (ab53104) and Rabbit anti-ErbB4 (ab32375), sourced from Abcam. For Western blot: fresh PFC tissue was quickly cut on ice, broken down in RIPA buffer with proteinase inhibitors, and prepared for SDS-PAGE. Mouse GAD67 (67648-1-Ig) and β-actin Monoclonal antibody (66009-1-Ig), from Proteintech; Rabbit Anti-HRG1 antibody (bs-1814R), from bioss; and Mouse HER4 Antibody (AF6445), from Affinity Biosciences. Esclorazepam was supplied by Jiangsu Hengrui Medicine Co., Ltd. The drug is dissolved in 0.9% saline solution and administered via intraperitoneal injection at a dose of 10 milligrams per kilogram of body weight.

### 2.4 Western blot

Following the behavioral tests, the mice were euthanized either 30 minutes or 24 hours post-injection, and their PFC tissues were promptly dissected and homogenized in lysis buffer obtained from Beyotime (China). The homogenate was then mixed with an equal volume of 5× loading buffer. The resulting protein mixture was separated using a 10% SDS-PAGE gel and transferred onto a nitrocellulose membrane. The membrane was blocked with 5% skimmed milk at room temperature for 2 hours. The primary antibody was diluted in a suitable solution (Dilution ratio:β-actin and GAD67 1:10000; ErbB4 and NRG1 1:1000) and incubated with the membrane overnight at 4 °C. Afterward, the membrane was thoroughly washed with TBST three times, each for 10 minutes. The secondary antibody, labeled with the same HRP as the primary antibody system, was diluted with 5% milk powder or BSA to the required ratio (1:10000) and incubated with the membrane at room temperature for 1 hour. Following incubation, the membrane was again washed thoroughly with TBST three times, each for 10 minutes. The NC membrane was then placed on an imager, and ECL working solution was added evenly before initiating exposure. The Western blot results were analyzed using Image J software to quantify the target protein. The ratio of grayscale values between each experimental group and the control group was used for statistical analysis, and the data was plotted using Graphpad Prism 8.

### 2.5 Immunofluorescence and imaging

We picked out the desired brain slices and placed them in a new 24-well plate, and washed the brain slices with PBS 3 times, each time lasting 5 minutes. Prepared the sealing liquid (PBS containing 10% NDS, 1% BSA with 0.3% TritonX-100), added the sealing liquid to the 24-well plate, removed the brain slice from the PBS, placed it in the sealing liquid, and sealed the brain slice at room temperature. The time of closure was usually 2 hours to ensure that the surface of the brain slice was completely closed to prevent nonspecific binding in subsequent steps. Prepared a primary antibody dilution (PBS containing 1% NDS, 1% BSA with 0.3% TritonX-100), diluted the primary antibody (dilution ratio 1:200) and added it to an 8-conjugate tube, completely covered the brain slice, placed it in a refrigerator at 4 °C and incubated it overnight to allow the antibody to bind to the target protein. Removed the brain slices and washed the brain slices using PBS 3 times for 15 min each to completely remove unbound primary antibody. Prepared a secondary antibody dilution (containing 1% NDS, 0.3% TritonX-100), diluted the secondary antibody (dilution ratio 1:200) and add it to a 24-well plate to completely cover the brain slice. Incubated for 1 hour at room temperature away from light. Finally, the brain slices were washed 3 times using PBS for 15 min each time. The brain slices were fixed on microscope slides using the bleaching method, and the operation was performed in a dark room. After evaporation of water, DAPI-containing anti-fluorescent attenuating sealer was added to the brain slices and the slices were sealed using coverslips. The sealed slides were placed in a storage box and waited for 5 minutes before observation using a fluorescence microscope. If observation and photography were not immediately possible, the brain slices were stored in a 4 °C refrigerator. After the photographs were taken, the brain slices were labeled and the fluorescence pictures were statistically analyzed using image J software.

### 2.6 Behavioral tests

#### 2.6.1 The chronic social defeat stress paradigm

The Chronic Social Defeat Stress (CSDS) modeling technique was implemented based on methods outlined in relevant literature (30). In rodents, repeated exposure to social defeat stress can induce a depressive-like phenotype marked by anhedonia and social avoidance. The modeling process involved two stages: initially, CD-1 mice were screened for aggressiveness over three days. CD-1 mice were chosen as attacker mice if they meet two criteria (1): They must successfully attack continuously for at least two times during each of the three 180-second screening sessions (2). Each initial attack delay must be less than 60 seconds, as per protocol requirements. CD-1 mice that did not meet these criteria were excluded. Following this, a 10-day chronic social defeat stress model was established. The experimental group of C57BL/6J mice underwent social defeat stress for 10 minutes each day for 10 consecutive days. After each daily attack, C57BL/6J mice were transferred to a compartment opposite the CD-1 mice and remained there for the remaining 24 hours. A new CD-1 mouse cage was used each day to prevent habituation. The control group was housed in pairs in the same cage, separated by a partition. After 10 days, all C57BL/6J mice were placed individually in standard cages with free access to food and water. Social interaction experiments were conducted 24 hours later.

#### 2.6.2 Social interaction test

The Social Interaction Test (SIT) was used to evaluate behavioral changes in mice following social defeat stress. Rodents were social creatures and tended to approach unfamiliar mice to establish social connections. However, mice exposed to social defeat stress often exhibited isolated and avoidance behaviors, which were indicative of depressive-like behavior. By comparing mice exposed to social defeat stress with control group mice, the duration of contact with unfamiliar mice in the social interaction area per unit time and the time spent in the corner area can indicate the degree of depression in the experimental group. The Anymaze software was used to record the time mice spent in the social interaction area and corner area. The social interaction ratio (SI) was calculated as the time spent in the social interaction area during the second stage divided by the time spent in the social interaction area during the first stage. Mice with an SI < 1 exhibited social avoidance and were considered sensitive, while mice with an SI > 1 exhibited social preference and were considered resistant.

#### 2.6.3 Forced swimming test

The animals were placed in water, where they struggled to escape but ultimately failed, entering a state of immobility over time. During this process, parameters indicative of depressive-like behaviors were observed and documented. The experimental apparatus consisted of a cylindrical container, 30cm tall with a 10cm diameter, filled with (22 ± 1) °C fresh water, which was replaced between each pair of animals. The experiment lasted 6 minutes, including a 2-minute acclimatization period, followed by a 4-minute immobilization period where time was recorded. Two individuals conducted behavioral assessments based on predefined scoring criteria, and the average score was calculated.

#### 2.6.4 Sucrose preference test

To evaluate if rodents have a pleasure deficit, we offered them two drinking options: sucrose solution and plain water, and monitored their preferences. The sucrose preference test was divided into two stages: acclimatization and testing. During acclimatization, mice were placed in individual cages with two bottles of 1% sucrose solution, positioned similarly and leak-free. After 24 hours, one bottle was switched to plain water. Following another 24-hour period of fasting and dehydration, the mice were given access to both plain water and 1% sucrose solution in randomized positions. After 12 hours, the bottles were weighed again to measure each mouse’s consumption of both solutions. The sucrose preference ratio was then calculated using the formula: sucrose preference ratio = (sucrose consumption)/(total liquid consumption) x 100%.

### 2.7 Intraperitoneal injection

We determined the necessary amount of esketamine for each mouse based on its weight, and dissolved this quantity in saline to reach the desired concentration. Used an electronic scale to precisely measure each mouse’s weight. Subsequently, calculated the individual dose for each mouse and administered it via intraperitoneal injection using sterile methods. Groups of male mice (6–8 per group) received either 10 mg/kg of esketamine or saline, and were euthanized either 30 minutes or 24 hours post-injection. The prefrontal cortex (PFC), a brain area associated with severe depression and antidepressant action, was collected and its protein concentration was assessed.

### 2.8 Statistical analysis

In this study, behavioral experimental data were gathered and initially analyzed utilizing the Anymaze system, developed by Stoelting in the USA, for animal behavior analysis. Images of NRG1 and ErbB4 immunofluorescence staining in the prefrontal cortex were obtained with an Olympus two-photon laser confocal microscope (FV-1200MPE). Western blot results were then quantified using Image J software. All experimental data were presented as mean ± SEM and visualized with Graphpad Prism 8 for statistical analysis. In the study, a two - way ANOVA was conducted. The factors included in the analysis were group (control, resilient, and susceptible) and treatment (saline and esketamine). The dependent variables were behavioral experiment results, levels of the immunofluorescence - targeted protein, and overall protein expression. A two-way ANOVA was performed to analyze the main effects of treatment and group, as well as their interaction. When a meaningful interaction was identified, basic effects analyses were carried out with Sidak correction to compare the treatment groups within each phenotype. Tukey’s honestly significant difference (HSD) test was used to perform *post-hoc* pairwise comparisons for significant main effects, with the aim of correcting for multiple comparisons. All statistical results showed statistical differences with *P* < 0.05.

## 3 Results

### 3.1 Induction of depressive-like behavior and protein expression changes in mouse prefrontal cortex by CSDS

Utilizing the CSDS model, mice were divided into a non-stress control group and a stress experimental group. Social contact experiments were conducted to measure the social interaction (SI) ratio of the mice. Stress mice with an SI ratio below 1 were classified as susceptible, while those with an SI ratio above 1 were deemed resilient.

Compared to the control group, susceptible mice exhibited significant differences in social ratio within the social interaction and corner areas, there was a significant difference in social ratio of the resilient group in the corner area compared to the control group and the depression group. (*P* < 0.01) (**Figures 1B, C**). Furthermore, susceptible mice demonstrated a notable increase in immobility duration during the forced swimming test (*P* < 0.05) (**Figure 1D**) and a significant reduction in sucrose preference (*P* < 0.05) (**Figure 1E**). After 10 days of chronic social defeat stress, susceptible mice displayed depressive-like behaviors such as anhedonia and social avoidance.

After CSDS modeling, susceptible mice and resilient mice showed a downregulation of NRG1 protein expression in the prefrontal cortex (PFC) compared to the control group (*P* < 0.05), with no significant change in ErbB4 and GAD67 protein expression (**Figures 1F–I**).

### 3.2 Changes in depressive-like behavior after 30 minutes of esketamine administration

**Figure 2:**
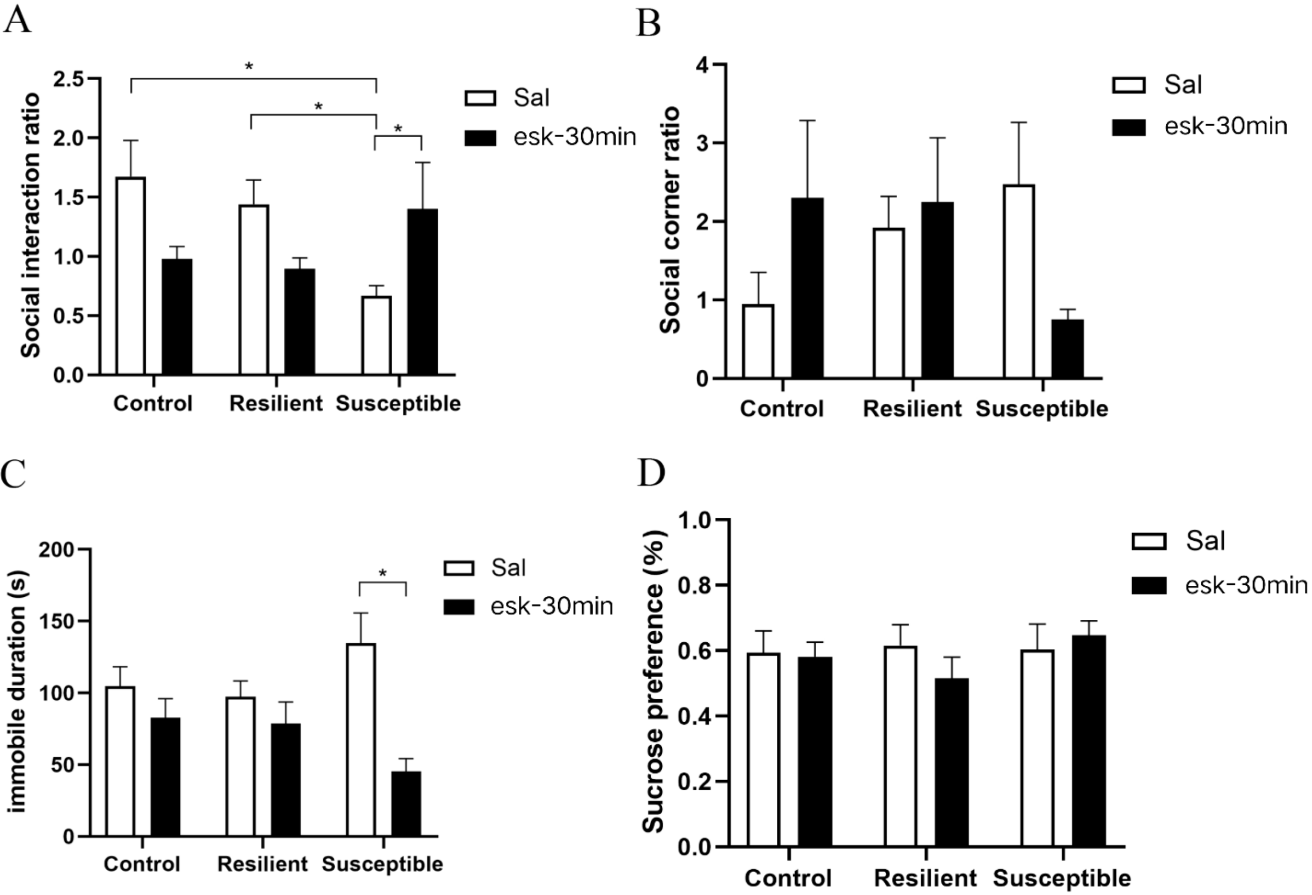
Behavioral experiment after 30 minutes of intraperitoneal injection of esketamine. **(A)** social interaction experiment, the social interaction ratio is the ratio of the activity time of C57BL/6J mice in the social contact area during the behavioral testing stage, with and without CD-1 mice; **(B)** the ratio of activity time in the corner area of C57BL/6J mice with and without CD-1 mice; **(C)** forced swimming experiment; **(D)** Sugar water preference experiment. (n: control + saline = 7, resilient + saline = 7, susceptible + saline = 6, control + esketamine = 6, resilient + esketamine = 8, susceptible + esketamine = 7). All data are expressed as mean ± SEM, *P < 0.05, two-way ANOVA With group (control/resilient/susceptible) and treatment (saline/esketamine) as independent variables, and the social interaction ratio, the corner social interaction ratio, immobility time during the forced swimming test and sucrose preference as the dependent variable, conducted a two-way analysis of variance, followed by any multiple comparisons test.

In this study, mice were intraperitoneally injected with esketamine, and their acute and sustained depressive-like behavioral responses were assessed. Acute responses were observed 30 minutes post-injection, while persistent responses were evaluated 24 hours post-injection. Group (control, resilient, susceptible) and treatment (saline, esketamine) were entered as factors in a two-way ANOVA with social-interaction ratio as the dependent variable. Main effects of group and treatment were non-significant, but a significant group × treatment interaction emerged [F(2, 32)= 5.61, *P* < 0.01]. The results indicated that intraperitoneal esketamine injection significantly affected the social interaction ratio of the saline-injected group. Among the three groups of mice saline-injected, the social interaction ratio differed significantly, the SI ratio followed the order: susceptible group < resilient group < control group (*P* < 0.05). Meanwhile, in susceptible mice, those injected with esketamine for 30 minutes had a significantly higher social interaction ratio compared to saline-injected mice (**Figure 2A**, P < 0.05). Two-way ANOVA revealed no significant main effects or interactions on either the corner social-interaction ratio or sucrose preference. while there was no significant difference in corner area social ratio (**Figure 2B**) and there was no significant decrease in sucrose preference (**Figure 2D**). Immobility time in the forced-swim test (FST) was analyzed by two-way ANOVA. A significant main effect of treatment was observed [F(1, 32)= 15.10, *P* < 0.001] and the immobility time in the forced-swim test was significantly longer in mice injected with saline than in those injected with esketamine. A group × treatment interaction was also present [F(2, 32)=4.12, *P* < 0.05]. *Post-hoc* tests revealed that esketamine markedly reduced FST immobility in susceptible mice (**Figure 2C**, P < 0.01).

### 3.3 Alterations in NRG1 and ErbB4 immunofluorescence after 30 minutes of esketamine administration

**Figure 3:**
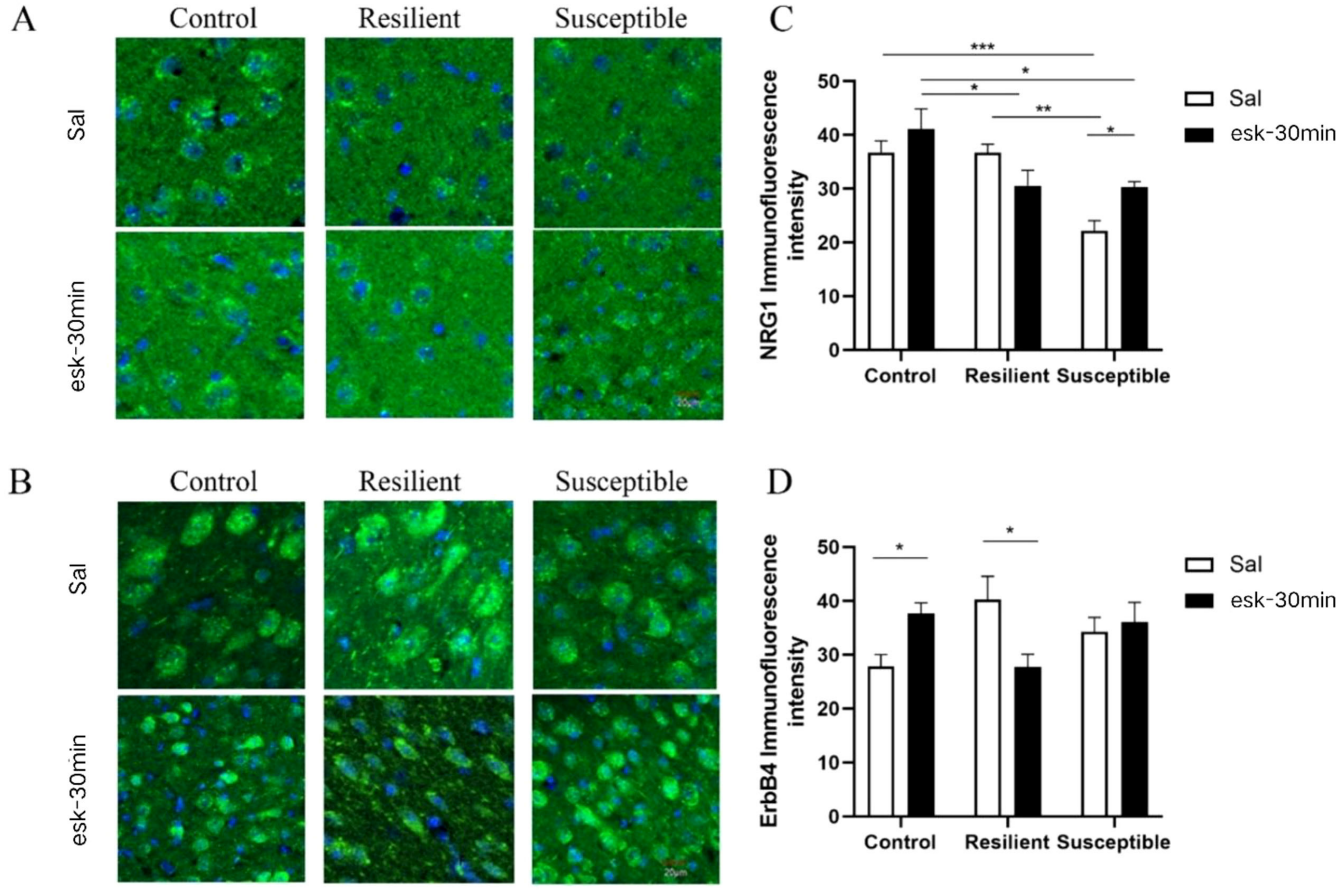
Immunofluorescence after 30 minutes of intraperitoneal injection of esketamine. **(A)** NRG1 immunofluorescence, blue: DAPI, green: FITC; **(B)** ErbB4 immunofluorescence, blue: DAPI, green: FITC; **(C)** NRG1 immunofluorescence quantification (n: control + saline = 8, resilient + saline = 5, susceptible + saline = 5, control + esketamine = 5, resilient + esketamine = 3, susceptible + esketamine = 4); **(D)** ErbB4 immunofluorescence quantification (n: control + saline = 4, resilient + saline = 4, susceptible + saline = 6, control + esketamine = 7, resilient + esketamine = 4, susceptible + esketamine = 6). All data are expressed as mean ± SEM, *P<0.05, **P<0.01, ***P<0.001 and the data is analyzed using two-way ANOVA with group (control/resilient/susceptible) and treatment (saline/esketamine) as independent variables, and the NRG1 and ErbB4 immunofluorescence as the dependent variable, conducted a two-way analysis of variance, followed by any multiple comparisons test.

NRG1 and ErbB4 levels in the mouse PFC were assessed using immunofluorescence technology. With group and treatment as independent variables, and NRG1 protein expression as the dependent variable respectively, conducted a two-way analysis of variance. Two -way ANOVA showed a significant main effect of group [F (2, 21) = 14.20, *P* < 0.001] and a significant group × treatment interaction [F (2, 21) = 3.89, *P* < 0.05]. The results revealed that compared to the control group and resilient group, NRG1 protein expression was significantly downregulated in the susceptible group injected with saline (*P* < 0.01). In susceptible mice, NRG1 protein expression increased after intraperitoneal esketamine injection (*P* < 0.05). Two-way ANOVA revealed a significant group × treatment interaction [F(2, 21)= 6.78, *P* < 0.01], In the control group, ErbB4 immunofluorescence was significantly higher in mice treated with esketamine compared to those treated with saline (P < 0.05). Conversely, in the resilient group, ErbB4 immunofluorescence was significantly lower in mice treated with esketamine compared to those treated with saline (*P* < 0.05, **Figure 3**).

### 3.4 Protein expression changes of NRG1, ErbB4, and GAD67 after 30 minutes of esketamine administration

**Figure 4:**
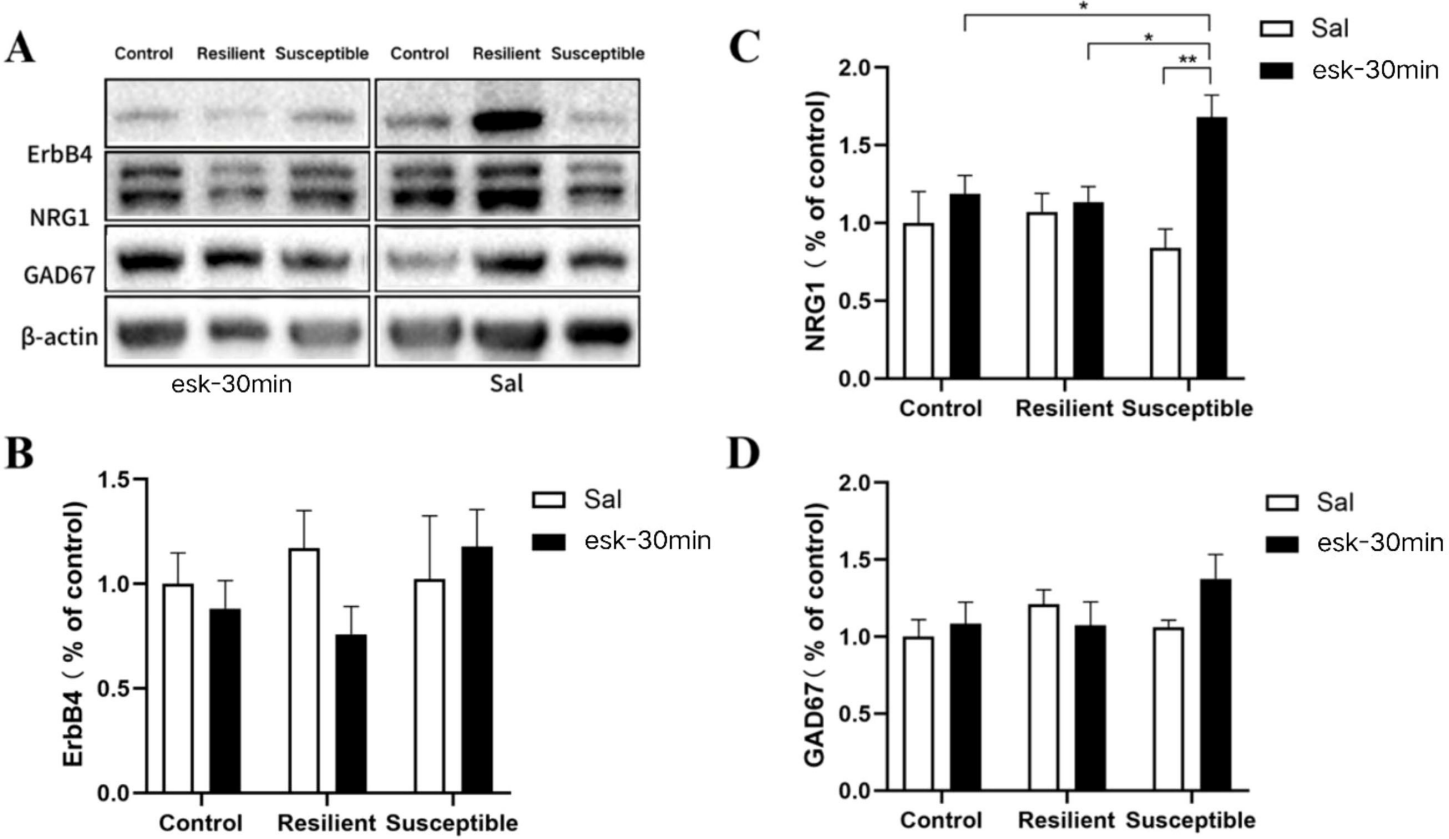
Changes in protein expression of intraperitoneal injection of esketamine. **(A)** Changes in protein expression of intraperitoneal injection of esketamine in WB results: 30 minutes after esketamine injection group and normal saline group from left to right, while from top to bottom are ErbB4, NRG1, GAD67, and β-actin; **(B)** There was no significant difference in the expression level of ErbB4 protein after 30 minutes of injection of a subanesthetic dose of esketamine (n: control + saline = 5, resilient + saline = 6, susceptible + saline = 4, control + esketamine = 5, resilient + esketamine = 6, susceptible + esketamine = 4); **(C)** The expression level of NRG1 in the PFC of susceptible group mice increased and was significantly higher than that of the other two groups after 30 minutes of injection of a subanesthetic dose of esketamine (n: control + saline = 6, resilient + saline = 7, susceptible + saline = 5, control + esketamine = 11, resilient + esketamine = 9, susceptible + esketamine = 9); **(D)** There was no significant difference in the expression level of GAD67 protein after 30 minutes of injection of a subanesthetic dose of esketamine (n: control + saline = 6, resilient + saline = 7, susceptible + saline = 5, control + esketamine = 11, resilient + esketamine = 8, susceptible + esketamine = 9). All data are expressed as mean ± SEM, with *P < 0.05 and **P < 0.01. two-way ANOVA with group (control/resilient/susceptible) and treatment (saline/esketamine) as independent variables, and the expression level of NRG1 and ErbB4 and GAD67 protein as the dependent variable, followed by any multiple comparisons test.

Changes in protein expression of intraperitoneal injection of esketamine. WB was used to detect NRG1, ErbB4, and GAD67 levels in the PFC of mice treated with esketamine for 30 minutes. Group and treatment served as independent variables in a two-way ANOVA with NRG1 protein expression as the dependent variable. Two -way ANOVA showed a significant main effect of treatment [F (1, 24) = 10.95, *P* < 0.01] and esketamine-injected mice displayed markedly higher NRG1 protein expression than saline- injected mice(*P* < 0.01).A significant group × treatment interaction emerged[F (2, 24) = 4.51, *P* < 0.05],*post-hoc* tests showed that esketamine elevated NRG1 levels in the PFC of susceptible mice relative to saline controls (*P* < 0.01), Among esketamine- injected mice, NRG1 was significantly higher in the susceptible group than in both the resilient and control groups. (**Figures 4A, C**, *P* < 0.05). Two-way ANOVA detected neither significant main effects nor interactions for ErbB4 or GAD67 protein expression, with no significant difference in ErbB4 and GAD67 protein expression (**Figures 4A, B, D**, *P* > 0.05).

### 3.5 Alleviation of CSDS-induced depressive-like behavior by 24 hours of esketamine administration

**Figure 5:**
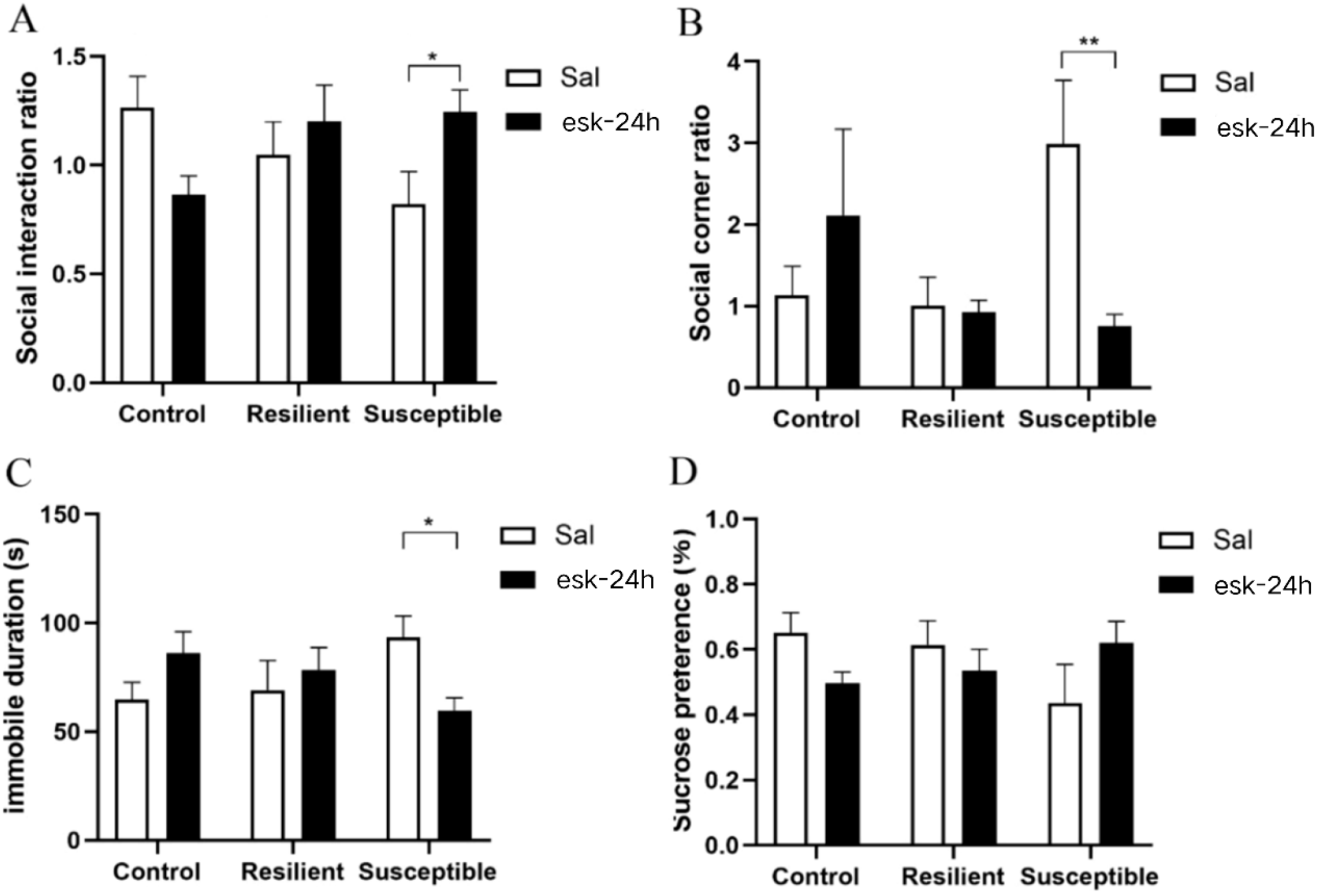
Behavioral experiment after 24 hours of intraperitoneal injection of esketamine. **(A)** social contact experiment, the ratio of activity time in the social contact area of C57BL/6J mice with and without CD-1 mice; **(B)** the ratio of activity time in the corner area of C57BL/6J mice with and without CD-1 mice; **(C)** forced swimming experiment; **(D)** Sugar water preference experiment. (n: control + saline = 7, resilient + saline = 7, susceptible + saline = 6, control + esketamine = 6, resilient + esketamine = 8, susceptible + esketamine = 7). All data are expressed as mean ± SEM, *P < 0.05, **P < 0.01. two-way ANOVA with group (control/resilient/susceptible) and treatment (saline/esketamine) as independent variables, and the social interaction ratio, the corner social interaction ratio, immobility time during the forced swimming test and sucrose preference as the dependent variable, followed by any multiple comparisons test.

Behavioral tests were performed 24 h after intraperitoneal esketamine injection to assess persistent responses. Group and treatment were entered as factors in a two-way ANOVA with the social-interaction ratio as the dependent variable. A significant group × treatment interaction emerged [F (2, 35) = 4.58, *P* < 0.05]. *Post-hoc* tests demonstrated that, in susceptible mice, those injected with esketamine for 24 hours had a significantly higher social interaction ratio compared to saline-injected mice (**Figure 5A**, P < 0.01). A two-way ANOVA with corner social-interaction ratio as the dependent variable revealed a significant group × treatment interaction [F (2, 35) = 4.96, *P* < 0.05]. *Post-hoc* tests showed that, 24h after esketamine injection, susceptible mice exhibited a significantly lower corner social-interaction ratio than after saline injection (**Figure 5B**, P < 0.01). A two-way ANOVA on forced-swim immobility time revealed a significant group × treatment interaction [F (2, 35) = 4.29, *P* < 0.05], *Post-hoc* tests showed that, 24 h after esketamine injection, susceptible mice exhibited significantly reduced immobility compared with saline-injected counterparts. (**Figure 5C**, P < 0.05). A two-way ANOVA with sucrose preference as the dependent variable indicated no significant main effects for either group or treatment, and no significant interaction effect was observed (**Figure 5D**).

## 4 Discussion

Depression is a mental health condition marked by persistent low mood, diminished interest in usual activities, and various physical and cognitive issues. The underlying causes, neurophysiological processes, and effective treatments and preventive measures remain poorly understood.

### 4.1 Inducing depressive like behavior in mice after CSDS modeling

Currently, the Chronic Social Defeat Stress (CSDS) model is a widely adopted tool in depression research, simulating stress factors through repeated social subordination (31). This model demonstrates predictive validity for treatment outcomes akin to those seen in patients (32), making it ideal for exploring neural and molecular mechanisms of depression and antidepressants (33). The stress-induced phenotypes, such as sensitivity and adaptability, mimic human depressive symptoms like social withdrawal, anhedonia, disinterest, hopelessness, and prolonged mood disturbance.

Our study revealed a 46% depression induction rate in mice using the CSDS model, lower than the literature-reported 60% to 70%. Genetic predisposition significantly influences an individual’s stress susceptibility and depressive-like behavior development, with experimental variations potentially affecting prevalence rates. Variables like cage dimensions, environmental conditions, social defeat duration and intensity, and timing of depressive-like behavior assessment can all skew results. And during the SI experiment, mice exposed to social failure stress may freeze in place after being placed in the box, so the data for this type of mouse were excluded. According to the standards of the CSDS paradigm, the SI ratio was calculated by dividing the time spent in the interaction zone with a CD1 mouse present by the time spent in the interaction zone without a CD1 mouse present. Mice that avoided the test completely (0 seconds) were labelled as susceptible (SI = 0). However, they were not included in the parametric analyses because the data did not meet the requirements for normality and variance homogeneity.

The susceptible group of mice exhibited social avoidance behavior. Social avoidance is the main risk factor for the development and maintenance of depression (34), prior studies indicate NRG1-deficient mice display reduced social novelty preference and altered social interactions (35). Interestingly, we found an increased interaction ratio in ket-30min mice compared to the saline group, without a corresponding corner ratio decrease. The mice seemed to spend more time in the arena’s center. The susceptible group’s prolonged immobility suggests heightened despair. The decreased sucrose preference ratio in these mice may indicate reward system abnormalities. These findings support the hypothesis that social failure triggers depressive phenotypes in susceptible mice. In **Figure 1**, the CSDS paradigm increased forced-swim immobility. By contrast, **Figure 2** shows no such increase. This discrepancy may arise from (i) a lower proportion of stress-susceptible individuals in the cohort examined in **Figure 2**, (ii) subtle procedural variations between experiments, or (iii) the sample size or statistical power in **Figure 2** may have been insufficient to detect a significant effect.

### 4.2 The mechanism of protein and fluorescence changes in the PFC

The expression of NRG1 in the prefrontal cortex (PFC) of susceptible mice decreased, while ErbB4 levels remained unchanged. It has been indicated by research that a significant role is played in the pathogenesis of depression by the NRG1-ErbB4 signaling pathway (36). Long-term memory decline in a mouse model of LPS-induced systemic inflammation is mediated by dysfunction of NRG1/ErbB4 signaling (37). Evidence has been provided in preclinical studies that depression-related white matter deficits are linked to disruptions in synaptic plasticity that are mediated by pathways. It was reported in the study that NRG1 signaling was downregulated and dendritic spine density was diminished in rodent models of depression (38). Research indicates an increase in NRG1 ubiquitination in susceptible mice, with ubiquitin ligase-mediated downregulation of NRG1 being pivotal in the depressive behavior of CSDS mice (39). Similarly, NRG1 levels were notably lower in the cortex of rats modeling postpartum depression, accompanied by a reduction in synaptic dendritic spines in the medial prefrontal cortex and nucleus accumbens. Past studies also support the downregulation of NRG1 expression (18).

Compared with the control group, there was no significant difference in the expression of GAD67 in PFC of susceptible group. GAD67 modulates neurotransmitter levels by converting glutamate to GABA, impacting neuronal excitability and inhibition. Early research indicates that GAD67 expression is lower in the PFC and hippocampus of individuals with MDD (40, 41). More recently, studies have demonstrated that chronic social defeat stress lowers the surface expression of GABAARs and disrupts GABAergic neurotransmission in the ventral hippocampus (42). Previous studies have shown that chronic stress leads to decreased GAD67 in the prefrontal cortex and hippocampus (43). However, we did not obtain corresponding results. Firstly, in terms of experimental design, it may be related to the duration of chronic social defeat stress that the mice we applied received every day. Our application time was 10 minutes, and even after using the longest time required per day, the number of depressed mice in the SI experiment is still relatively small, which may lead to insufficient sample size and low repetition rate.

### 4.3 The reasons and mechanisms of esketamine changing depressive like behavior

After 30 minutes and 24 hours of intraperitoneal esketamine injection, susceptible mice exhibited a marked reduction in the social-interaction ratio, immobility time shortened and depressive symptoms alleviated, highlighting esketamine’s swift impact on depression. While sucrose preference remained unchanged, this dissociation indicates that esketamine acutely modulates stress - induced social avoidance through mechanisms that are at least partially independent of those regulating hedonic responses. Firstly, it should be noted that the CSDS paradigm and esketamine dose (10 mg kg^-^¹, i.p.) employed here were within the range commonly reported in the literature (30, 44). Consequently, we cannot exclude the possibility that factors other than protocol itself—such as strain substrain differences, baseline sucrose preference, or subtle variations in defeat intensity—contributed to the absence of a measurable effect on sucrose preference in the present cohort. Future studies that systematically manipulate defeat duration and esketamine dosing will be required to clarify whether our specific experimental configuration uniquely influences sucrose preference. Moreover, the specific genetic background and prior experiences of the animals in our study cohort could have contributed to the divergent results (45). Esketamine, a potent NMDA receptor antagonist, binds to both synaptic and extrasynaptic NMDA receptors. While synaptic NMDAR proportion increases during development, a significant amount remains extrasynaptic in adulthood (44). The response triggered by NMDAR varies based on its location; synaptic NMDAR activation, primarily through calcium signaling, fosters neuroprotection, whereas extrasynaptic NMDAR activation promotes cell death. Research suggests that disrupted glial cell function under stress raises extracellular glutamate levels, activating extrasynatic NMDAR and causing cellular damage, leading to dysfunction and neurodegeneration (46). Decreased synaptic glutamate function can induce depressive-like behavior in animals. Chronic stress may impair glial excitatory amino acid transporter (EAAT) function, disrupting synaptic glutamate clearance, causing glutamate efflux, and enhancing extracellular glutamate receptor activation. Chronic stress and long-term glucocorticoid treatment may slow neurotransmitter circulation in the prefrontal cortex, reducing glutamate clearance relative to release. Repeated restraint or unpredictable stress can cause loss of AMPAR and NMDAR subunits on PFC neurons (47).

Esketamine’s antidepressant effect on GABA interneurons begins by blocking NMDAR, inhibiting interneuron activity (48), disinhibiting PFC glutamate release, rapidly increasing glutamate levels, activating AMPA receptors and postsynaptic membrane depolarization, and stimulating NMDAR and L-type calcium channels, initiating downstream protein synthesis and dendritic spine formation (49). Elevated calcium in dendritic spines is a crucial local circuit change driven by esketamine. Preclinical evidence suggests that antipsychotics have neuroprotective effects via the NRG1/ErbB4-PI3K/AKT/mTOR signaling pathway. This indicates that the sustained antidepressant effects of esketamine may also involve mTOR-mediated synaptic restoration (50). In susceptible mice, 30 minutes of esketamine treatment increased NRG1 expression in the PFC, potentially due to esketamine’s NMDAR antagonism altering signaling pathways and impacting NRG1-related pathways. In the NRG1-ErbB4 pathway, NRG1 binding to the ErbB4 receptor initiates intracellular signaling, enhancing protein synthesis and synaptic formation. The context-dependent effects of esketamine on ErbB4 signaling highlight the complexity of the NRG1/ErbB4 pathway in stress modulation (51, 52). The differential response observed in the control and resilient groups indicates that the baseline state of the animals modulates the impact of pharmacological interventions. The therapeutic specificity of esketamine for susceptible mice is likely due to the restoration of the GABA-glutamate balance in the medial prefrontal cortex (mPFC), whereas resilient mice show no further behavioral change despite modest ErbB4 activation. This elucidates the rationale behind the pathway’s indispensability, though it is not a panacea for antidepressant action in non-pathological states (36). Our This highlights the necessity of accounting for individual differences when examining the effects of stress and potential treatments. Thus, esketamine may bolster this signaling pathway by upregulating NRG1-ErbB4 in the PFC, ultimately promoting protein synthesis.

Previous research indicates that ketamine regulates the excitatory/inhibitory (E/I) balance in cortical circuits, potentially initiating neural plasticity in the dorsomedial prefrontal cortex. Evidence suggests that GABA alterations contribute to ketamine’s response by restoring E/I balance in the PFC through homeostatic adaptations, facilitating its antidepressant effect (53). Study showed that there was no significant difference in the expression of GAD67 protein in the prefrontal cortex of susceptible mice after 30 minutes of sub-anesthetic esketamine. This may be influenced by various factors such as time, individual differences, adaptive responses, neural plasticity, and experimental design. The antidepressant effect of ketamine is usually related to its impact on neural plasticity. Research has shown that esketamine can rapidly activate neuroplasticity processes in PFC, but these changes may not be reflected solely through short-term expression changes of GAD67. Other signaling pathways, gene expression, or neuronal remodeling may play important roles in the subsequent time window (3).

Our findings may further elucidate the interaction between esketamine and the nervous system, offering new insights into its pharmacological effects. While this study focuses on the PFC, esketamine affects multiple brain regions, including the lateral habenular nucleus, which may contribute to its antidepressant effects. Further experiments are needed to clarify the mechanisms and biological significance of these changes.

### 4.4 Limitation

The experiment can only hypothesize about how esketamine influences the NRG1-ErbB4 pathway and alleviates depressive-like behavior in mice, lacking direct evidence to establish a causal link between them. Due to ethical and cost constraints, molecular assays were conducted with small sample sizes per group. While these findings have been powered to detect large effect sizes, it is essential that they are replicated in larger cohorts in order to validate the robustness and generalizability of the parametric estimates. Additionally, this research relied solely on two behavioral metrics to assess esketamine’s impact on depressive-like behavior in mice. And at the 24-hour mark after the injection of esketamine, only behavioral results were obtained, but no measurements of the molecular changes over the 24-hour period were made. Furthermore, it focused exclusively on the NRG1-ErbB4 pathway, our experimental design did not include the use of an NRG1/ErbB4 pathway antagonist (e.g., spironolactone) to validate the behavioral effects of esketamine, while intraperitoneal administration of esketamine might also affect various other signaling pathways and mechanisms. To test whether NRG1–ErbB4 signaling is required for the sustained effects of esketamine, future experiments will pretreat mice with the ErbB4-selective antagonist NVP-AUY922 (10 mg/kg, i.p.) before administering esketamine, after which the same behavioral and molecular endpoints will be assessed. We also recognize that intraperitoneal esketamine may act in different ways; therefore, we will measure the changes in proteins in the body (phosphoproteomic profiling) to understand how it works and to see the effect of NRG1–ErbB4 compared to other processes.

## 5 Conclusion

Esketamine may have a regulatory effect on the NRG1-ErbB4 signaling pathway in the frontal lobe of mice. Esketamine is involved in improving depression like behavior induced by chronic social frustration stress in mice.

## Data Availability

The original contributions presented in the study are included in the article/**Supplementary Material**. Further inquiries can be directed to the corresponding authors.
