# Supplementary figures and images for: Esketamine ameliorates depression-like behavior in mice via modulation of the NRG1–ErbB4 pathway
Source: Front Psychiatry. 2026 Apr 7;17:1722336. doi: 10.3389/fpsyt.2026.1722336 (PMC13095719; doi:10.3389/fpsyt.2026.1722336)

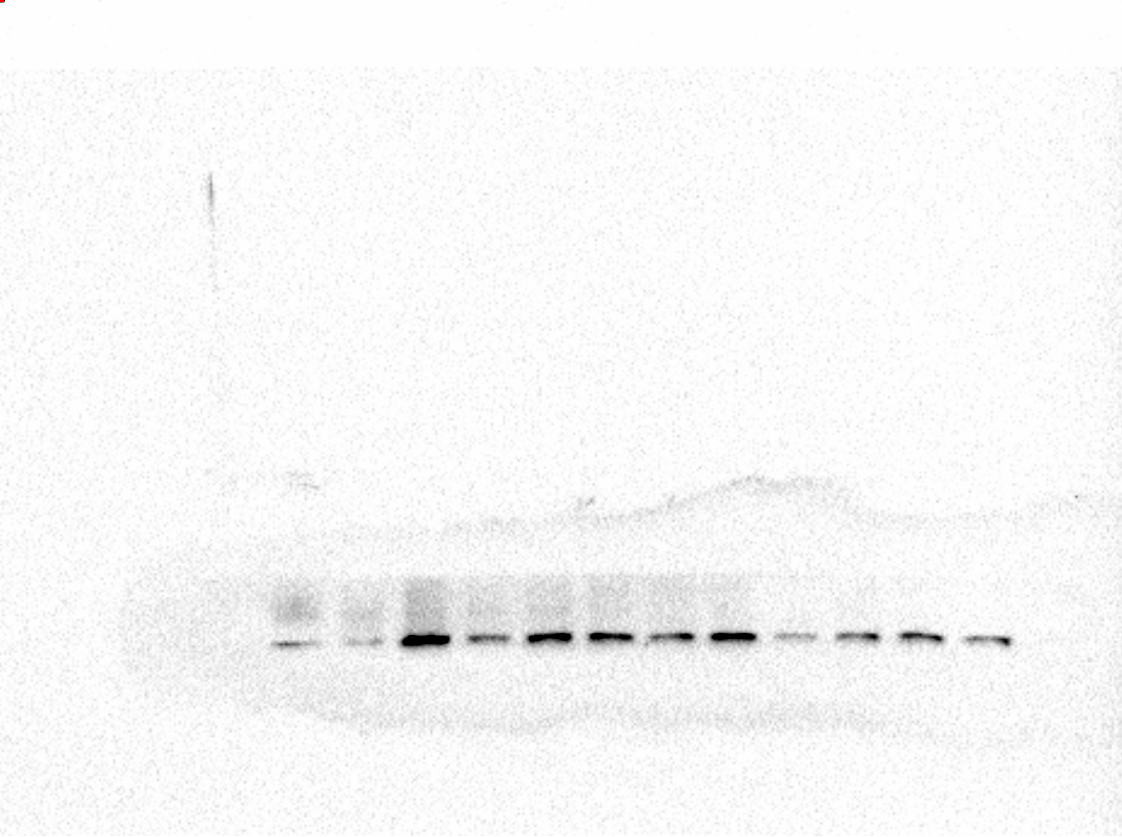

Supplement: Supplementary file 1 [file DataSheet1.zip › WB Images/ErbB4(intervened)/2023-10-11 140.tif]

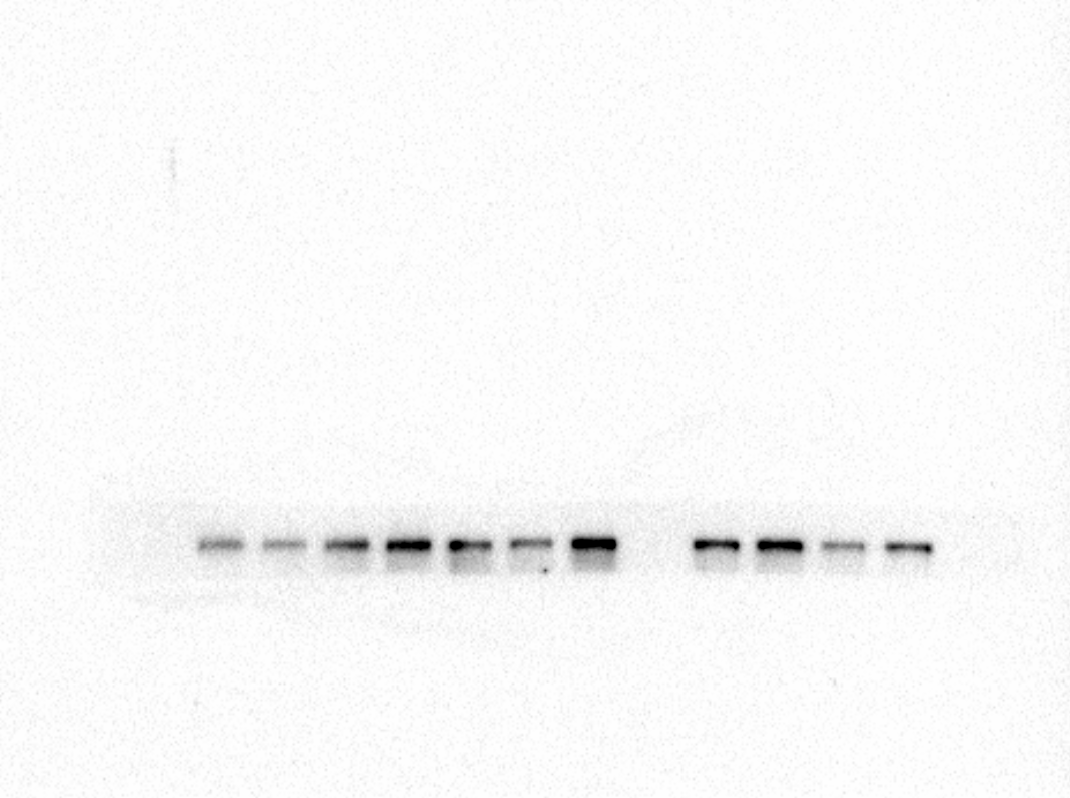

Supplement: Supplementary file 1 [file DataSheet1.zip › WB Images/ErbB4(intervened)/Administrator 2023-05-24 12 时 21 分_Exposure_49.5sec.tif]

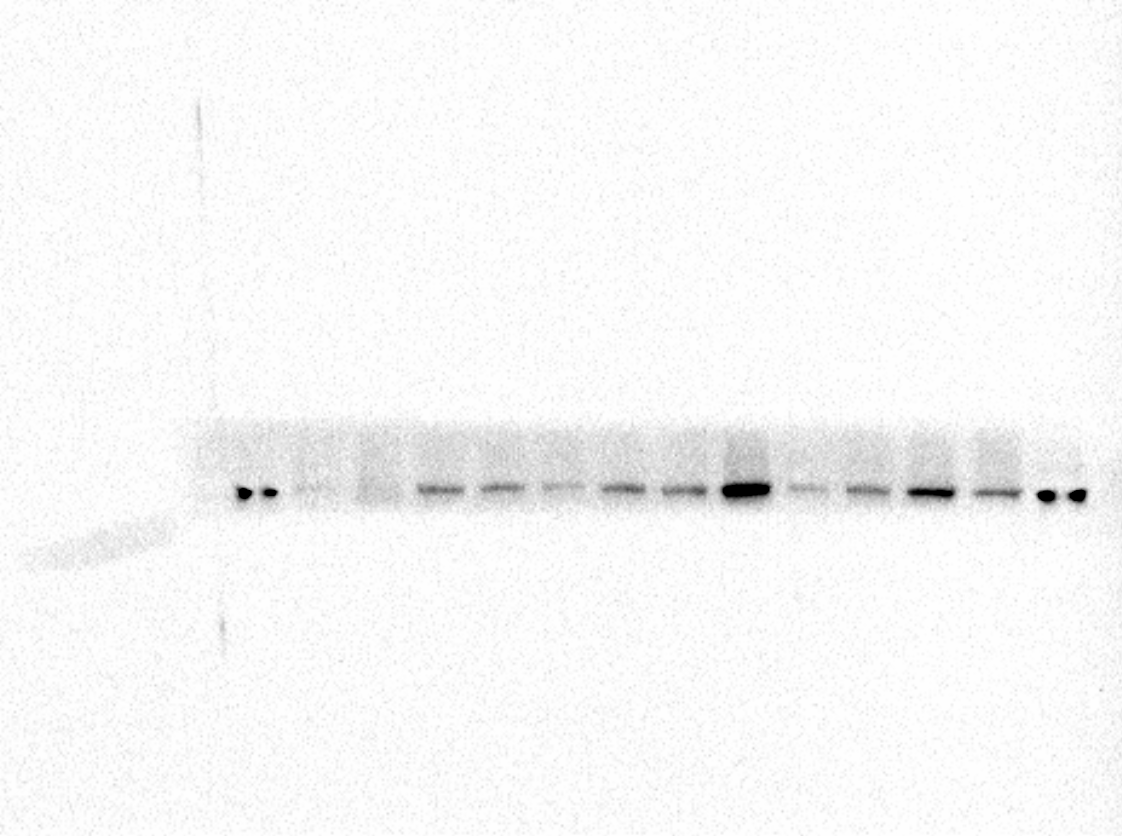

Supplement: Supplementary file 1 [file DataSheet1.zip › WB Images/ErbB4(intervened)/Administrator 2023-10-19 16 时 23 分_Exposure_120.0sec.tif]

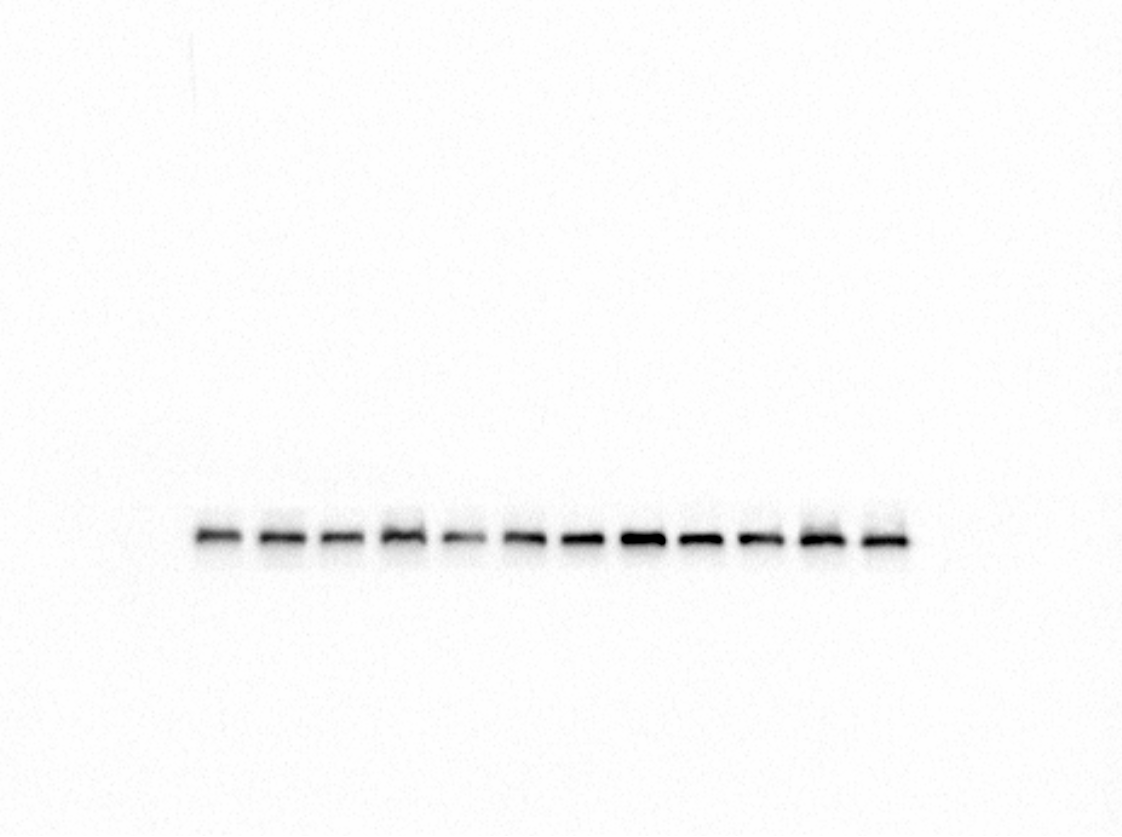

Supplement: Supplementary file 1 [file DataSheet1.zip › WB Images/ErbB4(intervened)/Administrator 2024-03-26 16 时 40 分_Exposure_49.6sec.tif]

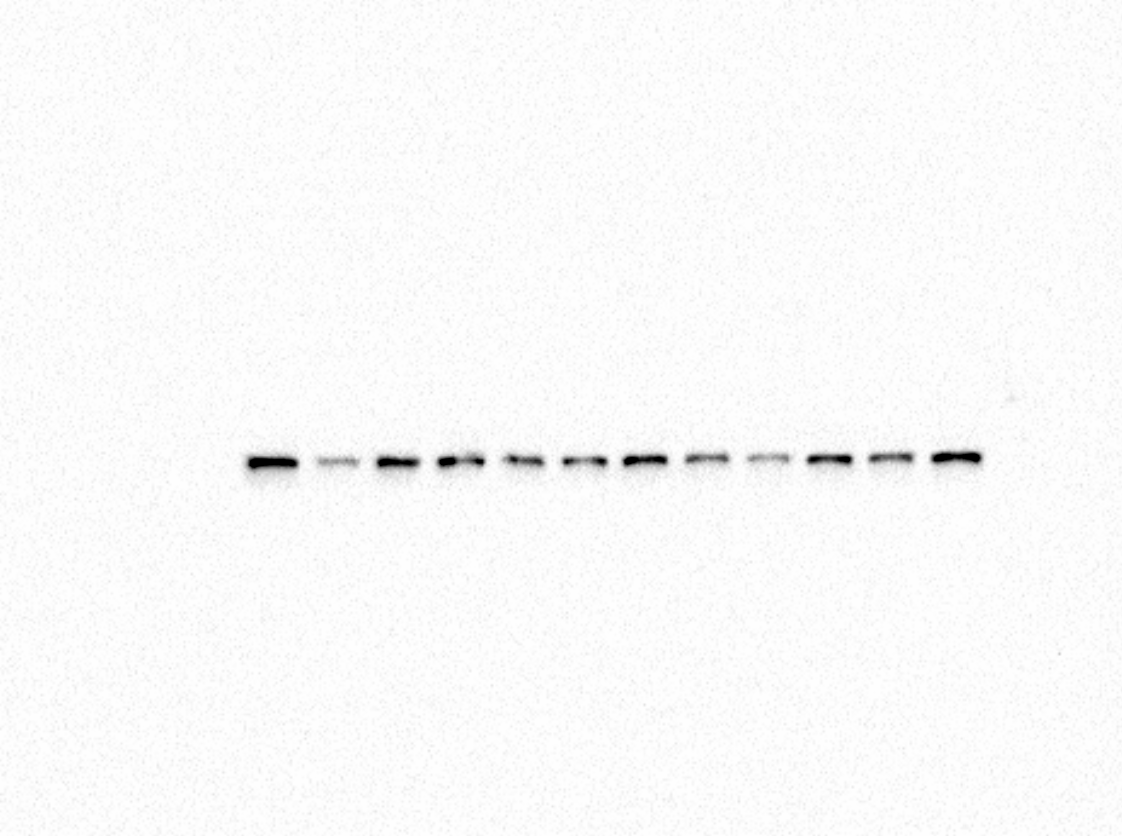

Supplement: Supplementary file 1 [file DataSheet1.zip › WB Images/ErbB4(without intervention)/20230303-1-140-Administrator 2023-03-03 17 时 42 分_Exposure_7.2sec.tif]

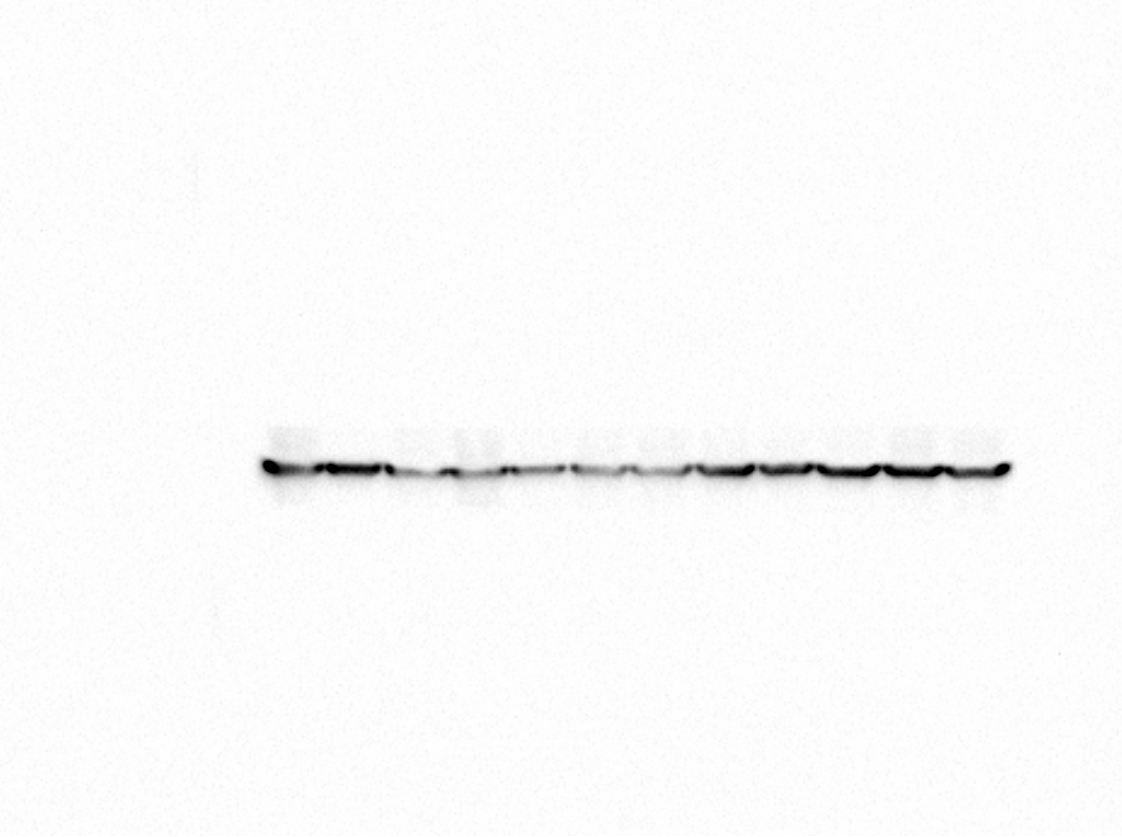

Supplement: Supplementary file 1 [file DataSheet1.zip › WB Images/ErbB4(without intervention)/20230303-1-43 时 55 分_Exposure_13.3sec.tif]

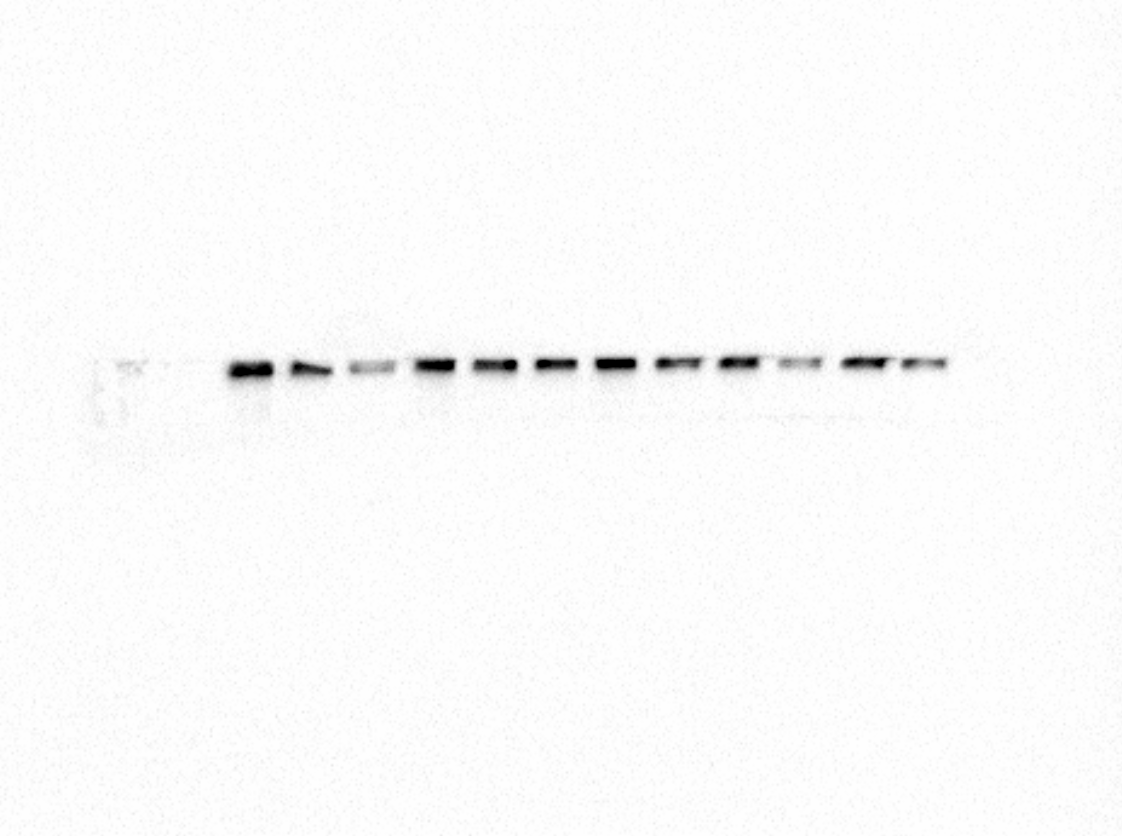

Supplement: Supplementary file 1 [file DataSheet1.zip › WB Images/ErbB4(without intervention)/20230307-140-Administrator 2023-03-07 11 时 55 分_Exposure_40.4sec.tif]

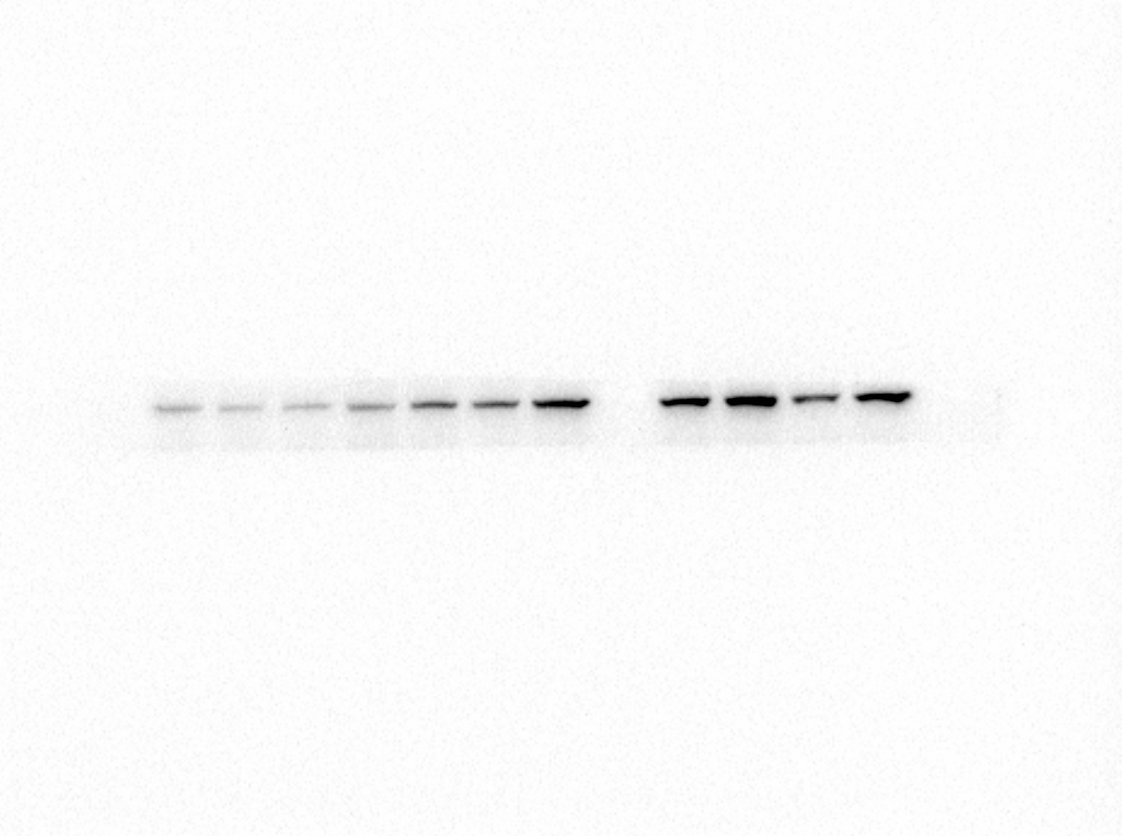

Supplement: Supplementary file 1 [file DataSheet1.zip › WB Images/GAD67(intervened)/Administrator 2023-05-24 11 时 39 分_Exposure_142.3sec.tif]

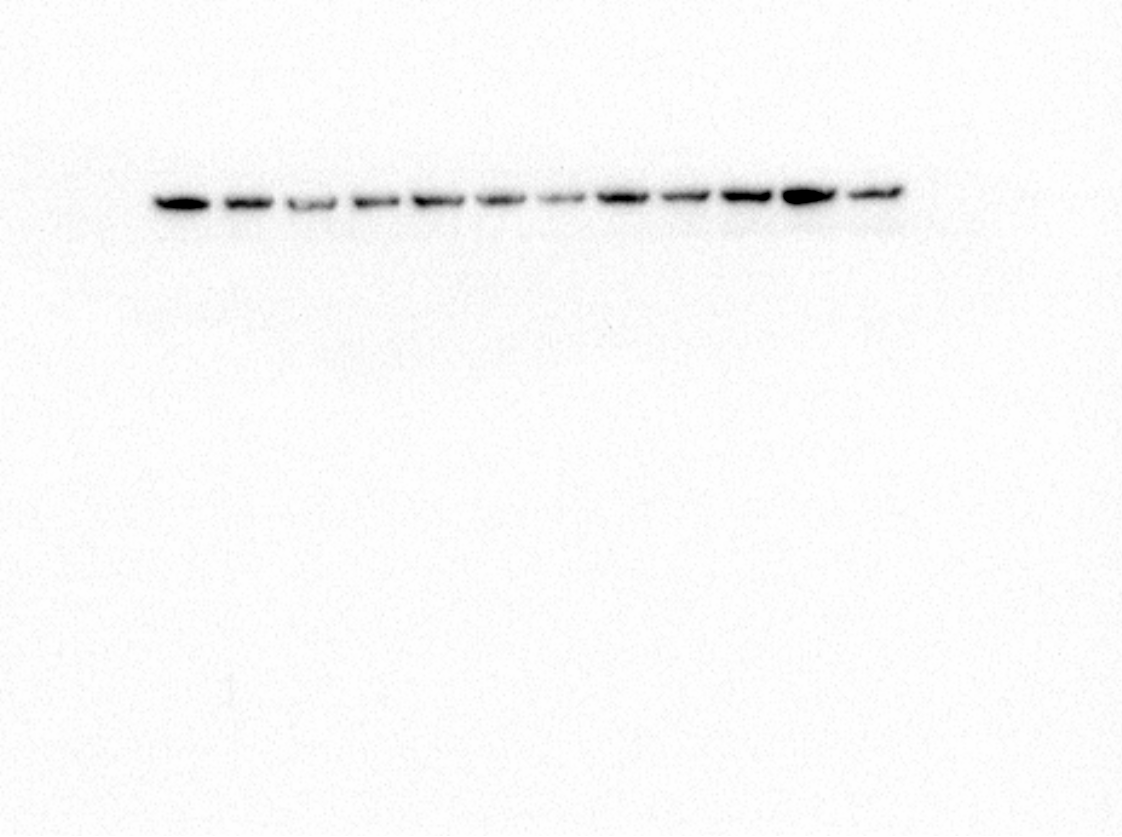

Supplement: Supplementary file 1 [file DataSheet1.zip › WB Images/GAD67(intervened)/Administrator 2023-10-11 16 时 24 分_Exposure_19.6sec.tif]

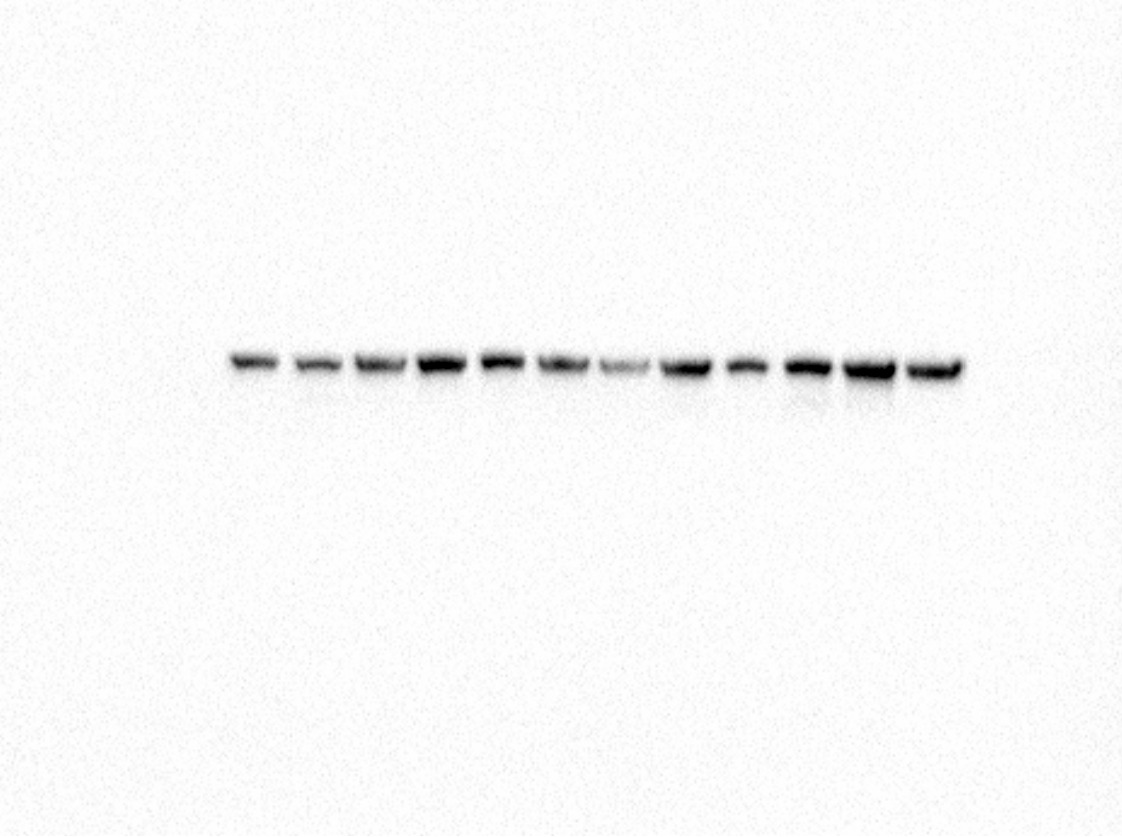

Supplement: Supplementary file 1 [file DataSheet1.zip › WB Images/GAD67(intervened)/Administrator 2023-10-19 16 时 16 分_Exposure_9.0sec.tif]

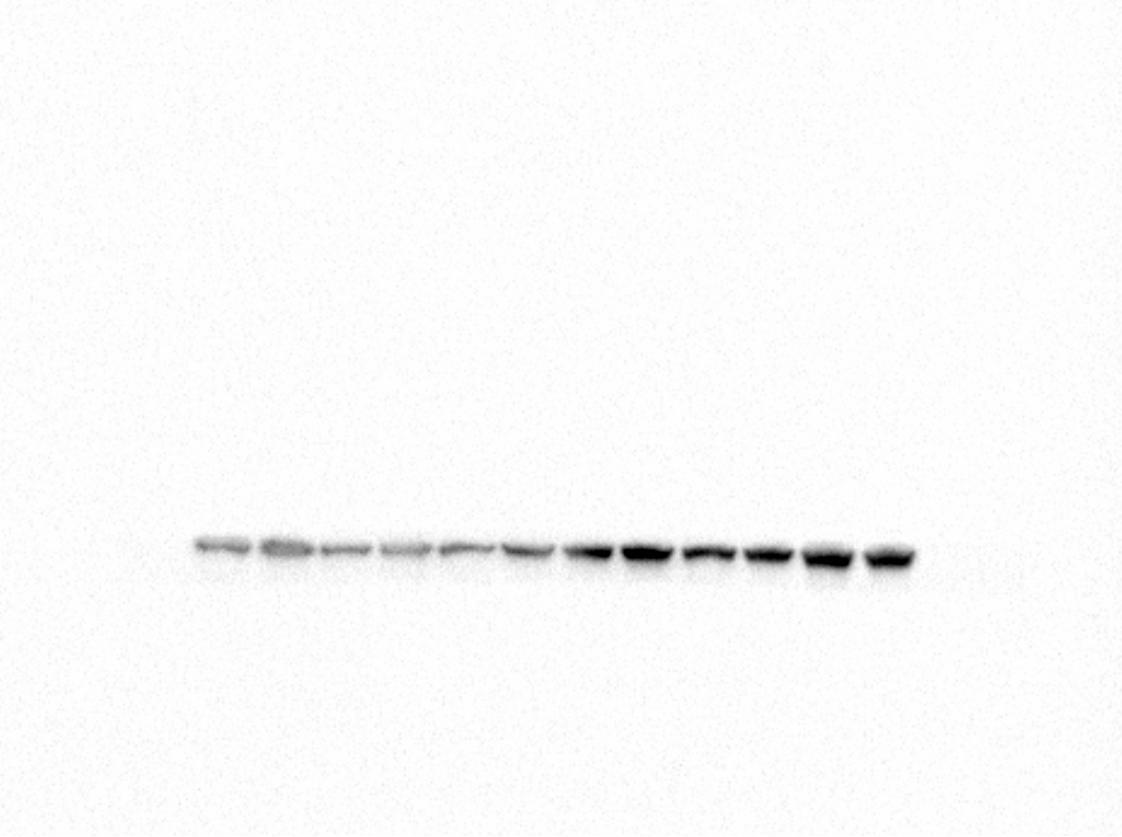

Supplement: Supplementary file 1 [file DataSheet1.zip › WB Images/GAD67(intervened)/Administrator 2024-03-26 16 时 49 分_Exposure_31.3sec.tif]

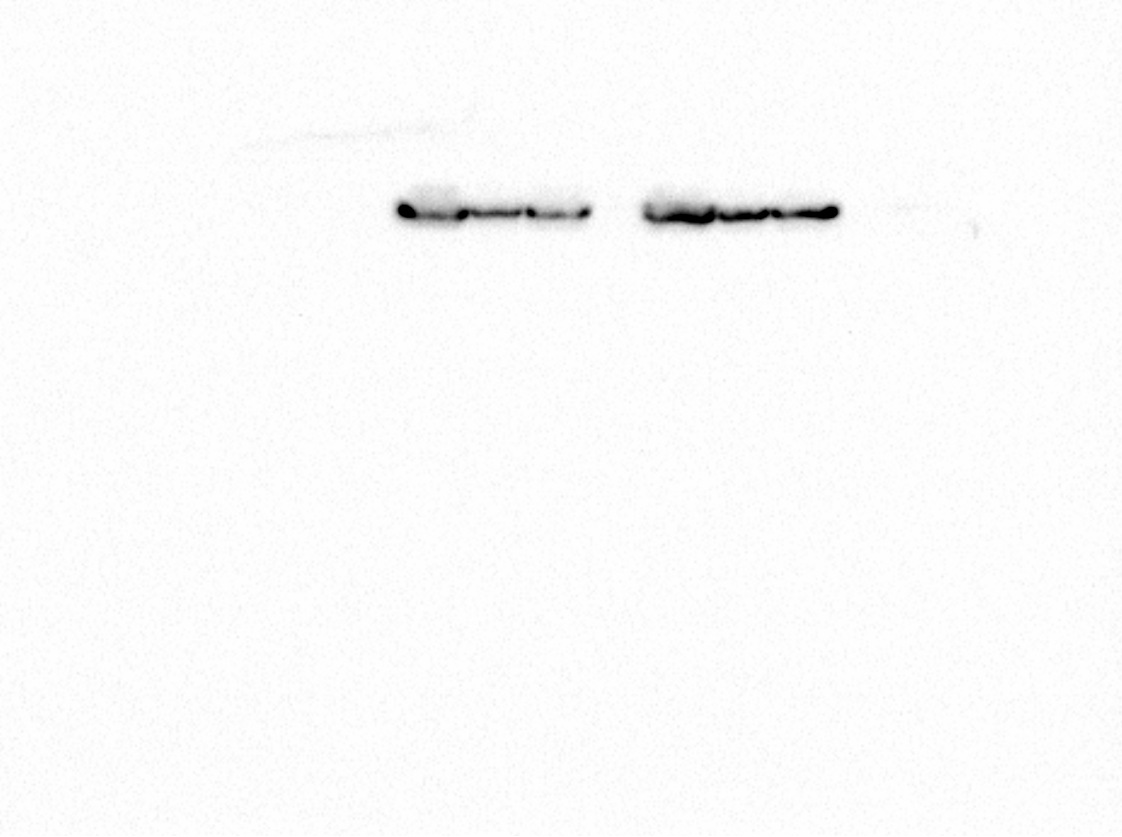

Supplement: Supplementary file 1 [file DataSheet1.zip › WB Images/GAD67(without intervention)/20230303-67xposure_31.9sec.tif]

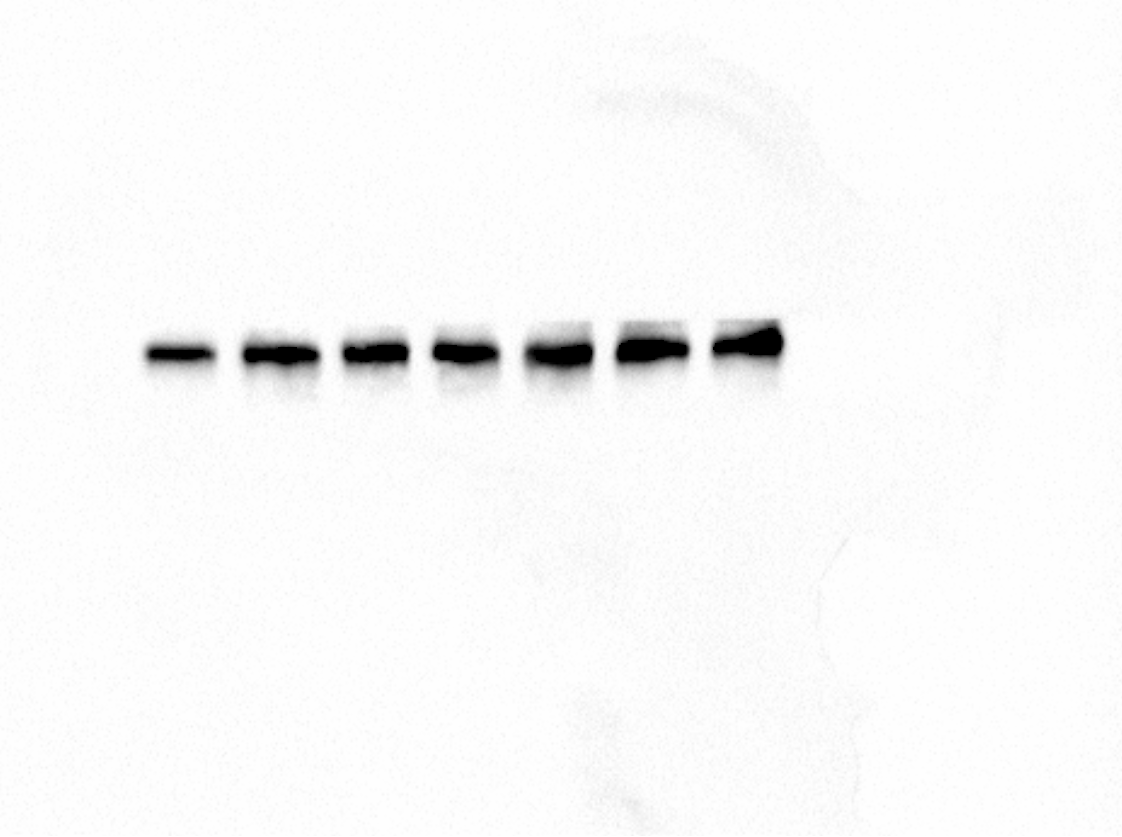

Supplement: Supplementary file 1 [file DataSheet1.zip › WB Images/GAD67(without intervention)/Administrator 2022-11-26 16 时 17 分_Exposure_58.0sec.tif]

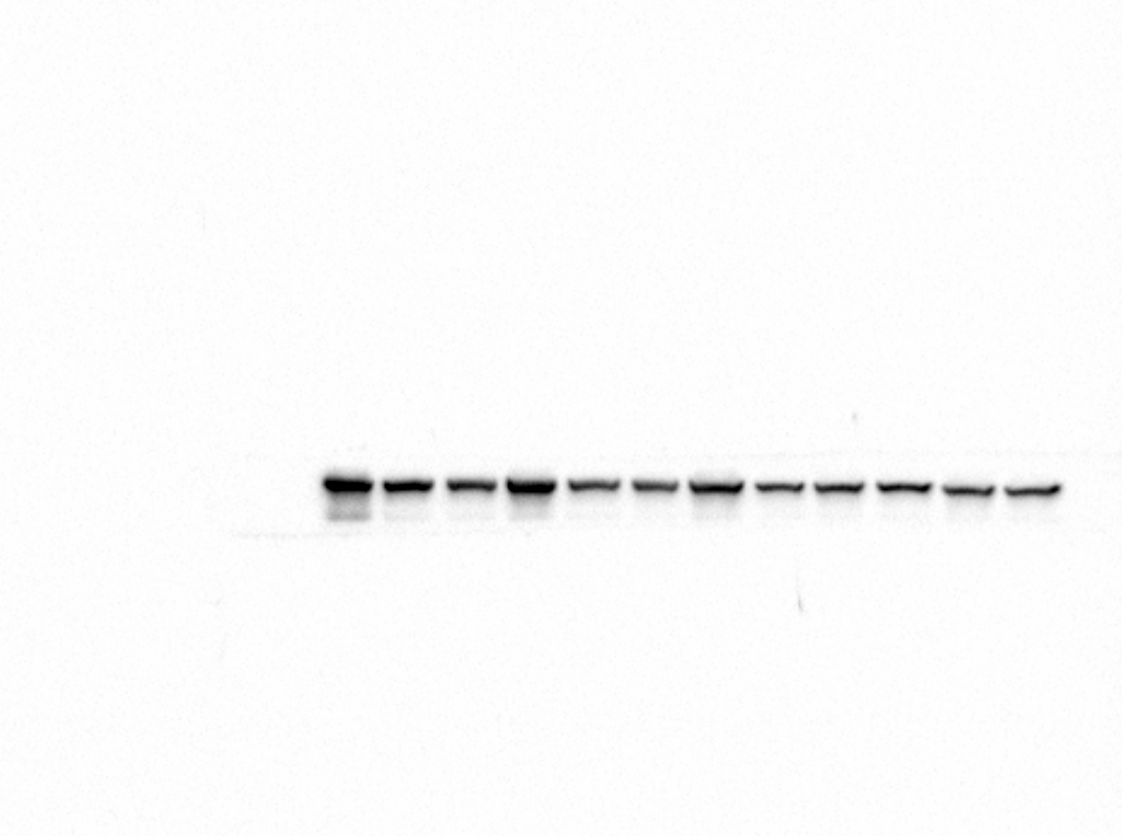

Supplement: Supplementary file 1 [file DataSheet1.zip › WB Images/GAD67(without intervention)/Administrator 2023-03-07 11 时 27 分_Exposure_19.2sec.tif]

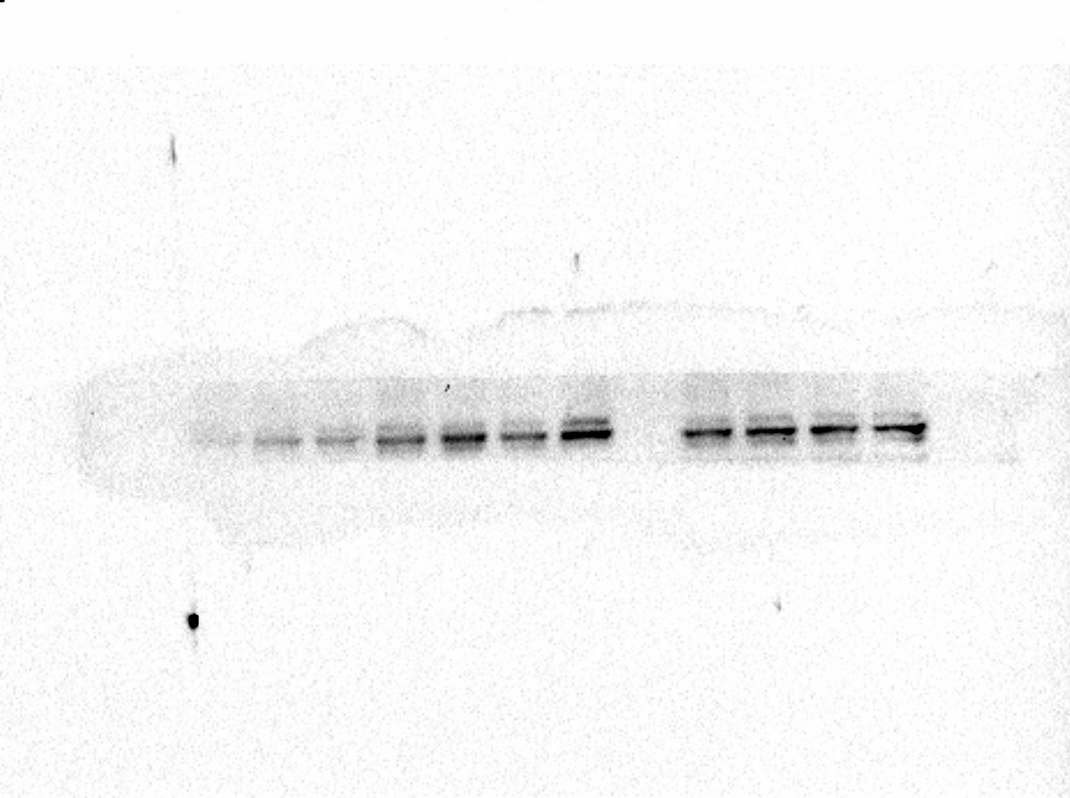

Supplement: Supplementary file 1 [file DataSheet1.zip › WB Images/NRG1(intervened)/105Administrator 2023-05-24 12 时 16 分_Exposure_128.4sec.tif]

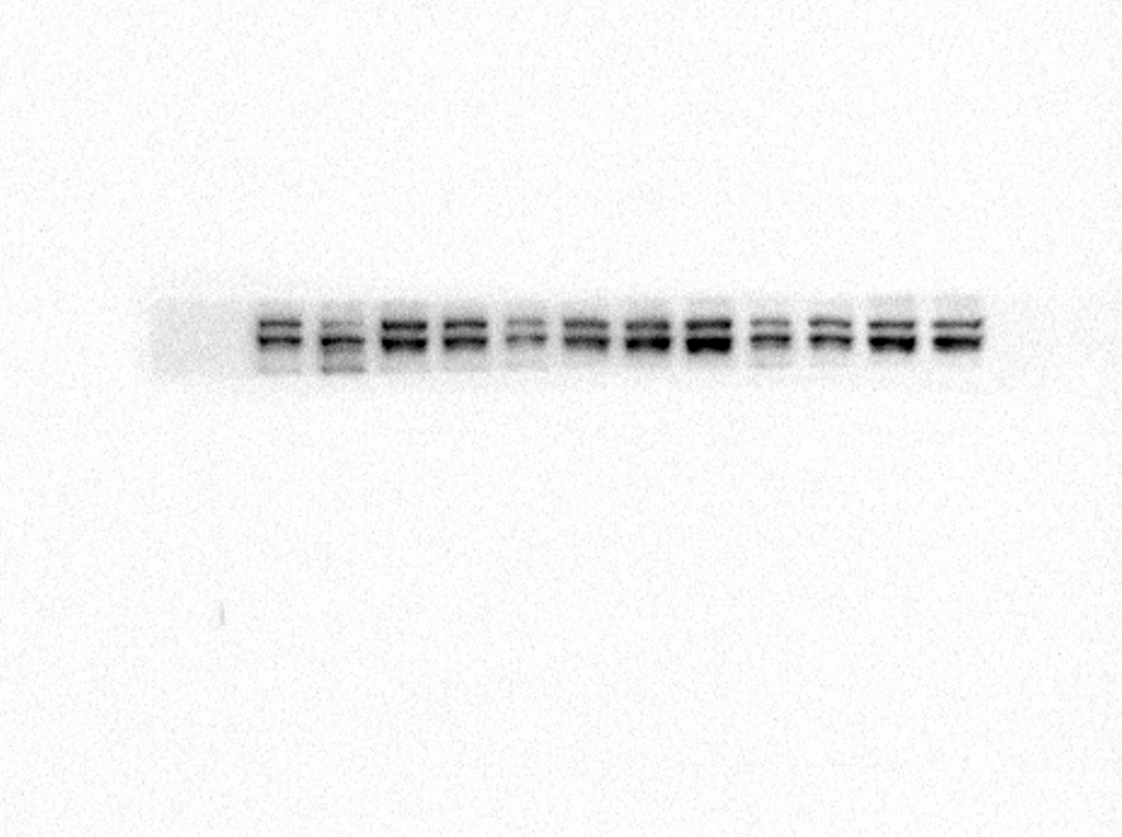

Supplement: Supplementary file 1 [file DataSheet1.zip › WB Images/NRG1(intervened)/2023-10-19 105.tif]

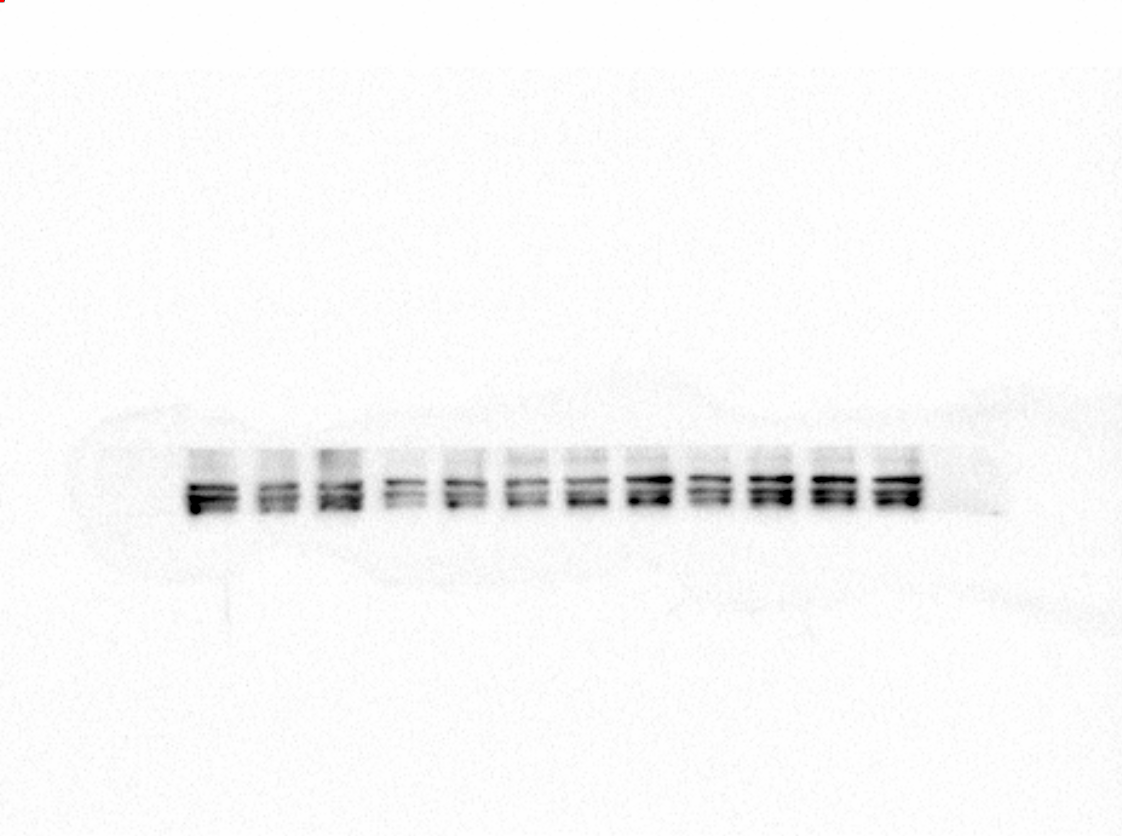

Supplement: Supplementary file 1 [file DataSheet1.zip › WB Images/NRG1(intervened)/20231011NRG1.tif]

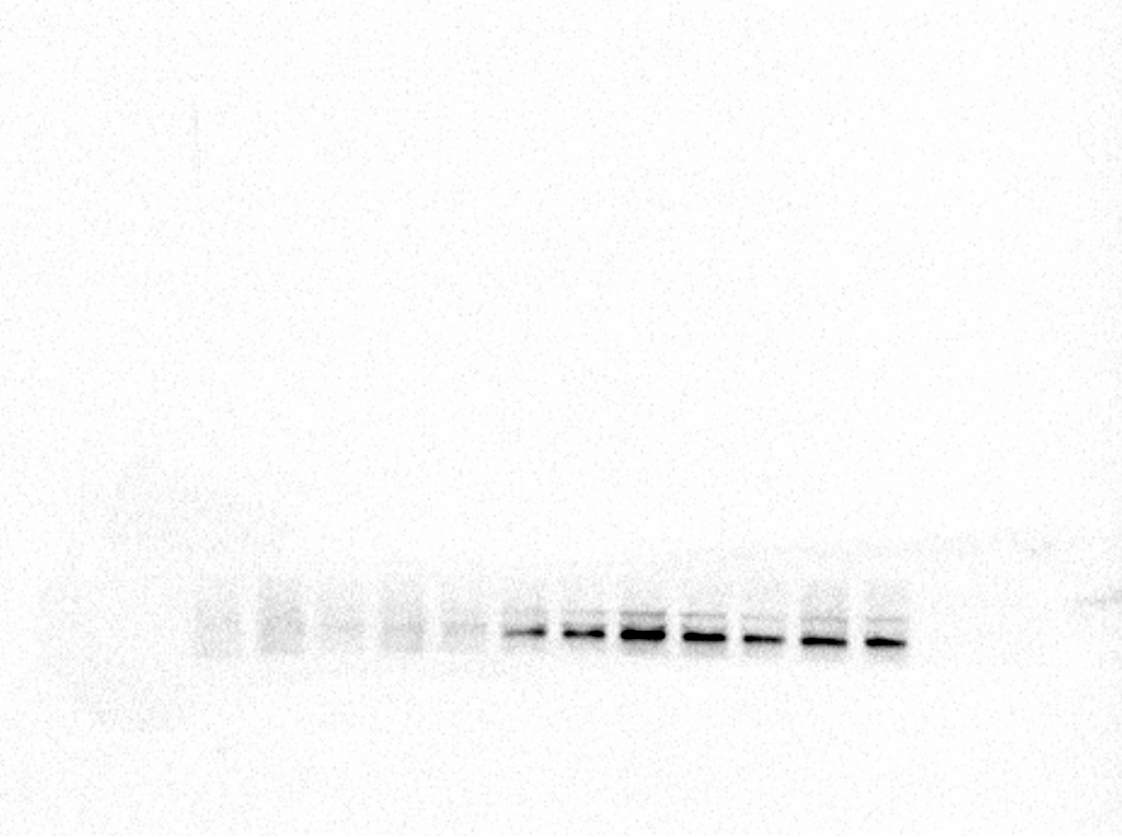

Supplement: Supplementary file 1 [file DataSheet1.zip › WB Images/NRG1(intervened)/2024-03-26 105.tif]

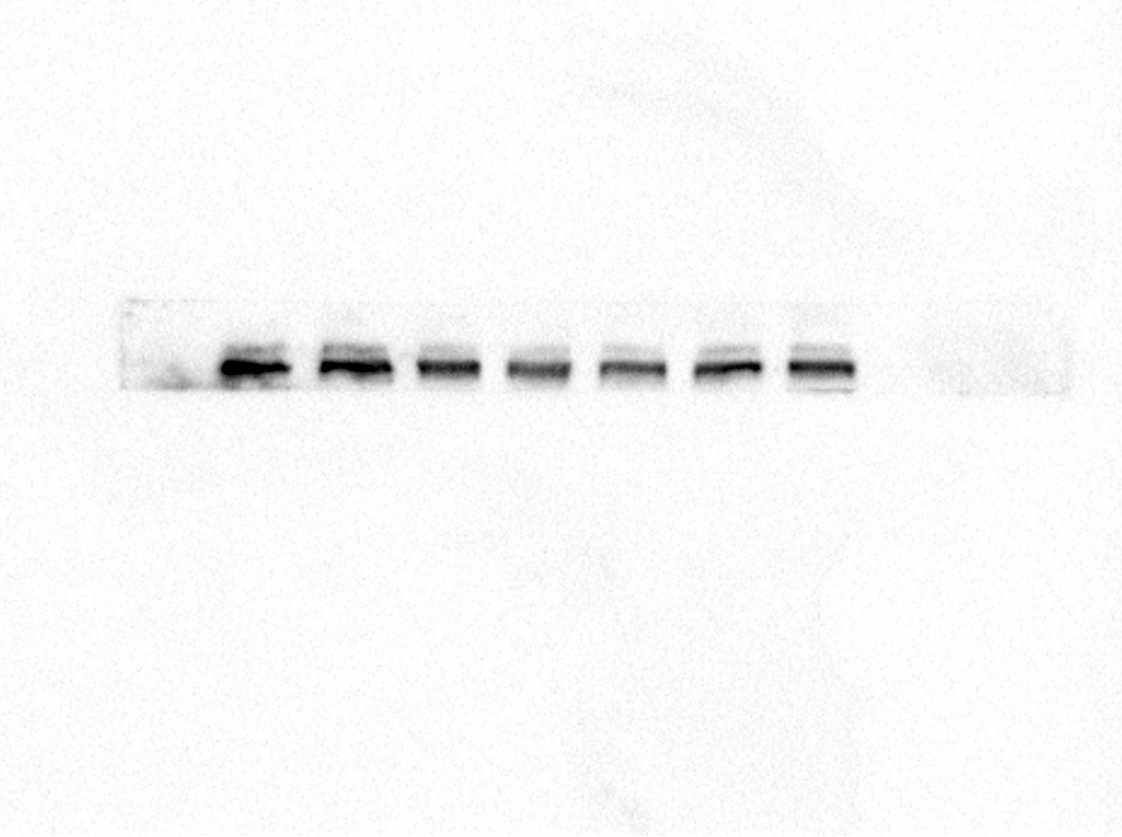

Supplement: Supplementary file 1 [file DataSheet1.zip › WB Images/NRG1(without intervention)/20221126-105.tif]

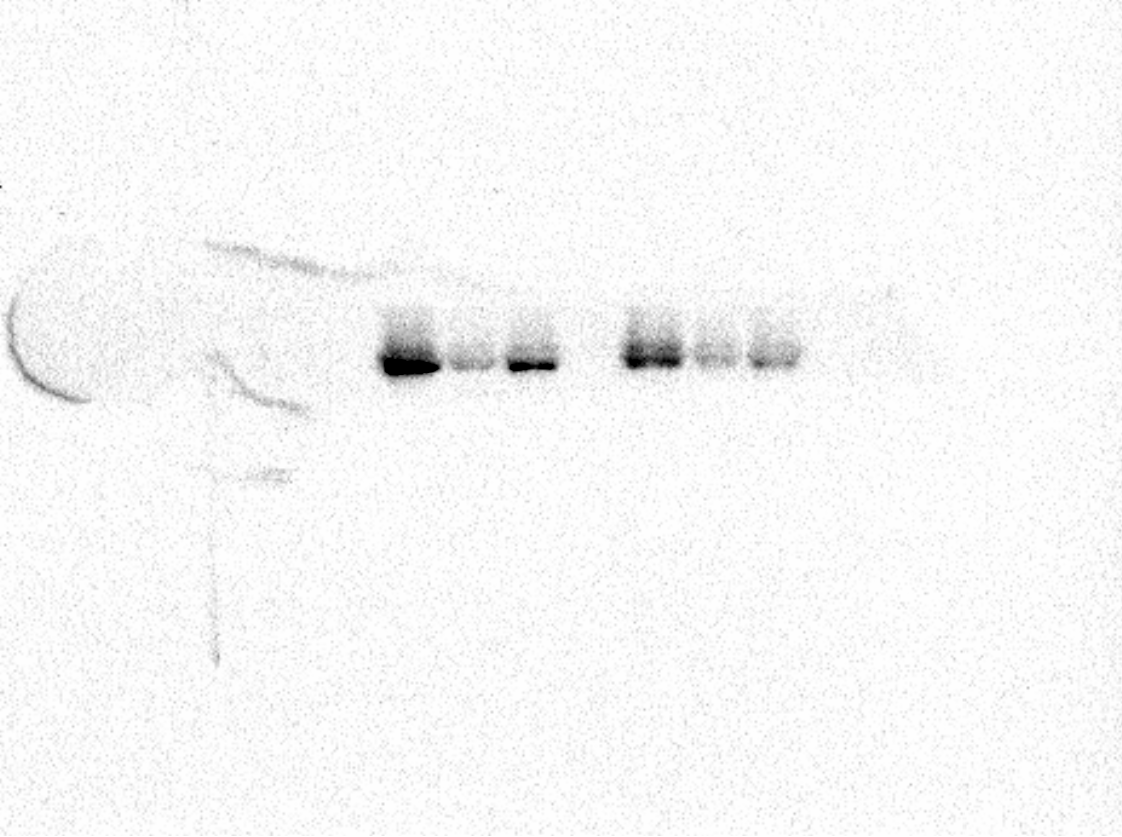

Supplement: Supplementary file 1 [file DataSheet1.zip › WB Images/NRG1(without intervention)/20230303-六个-105.tif]

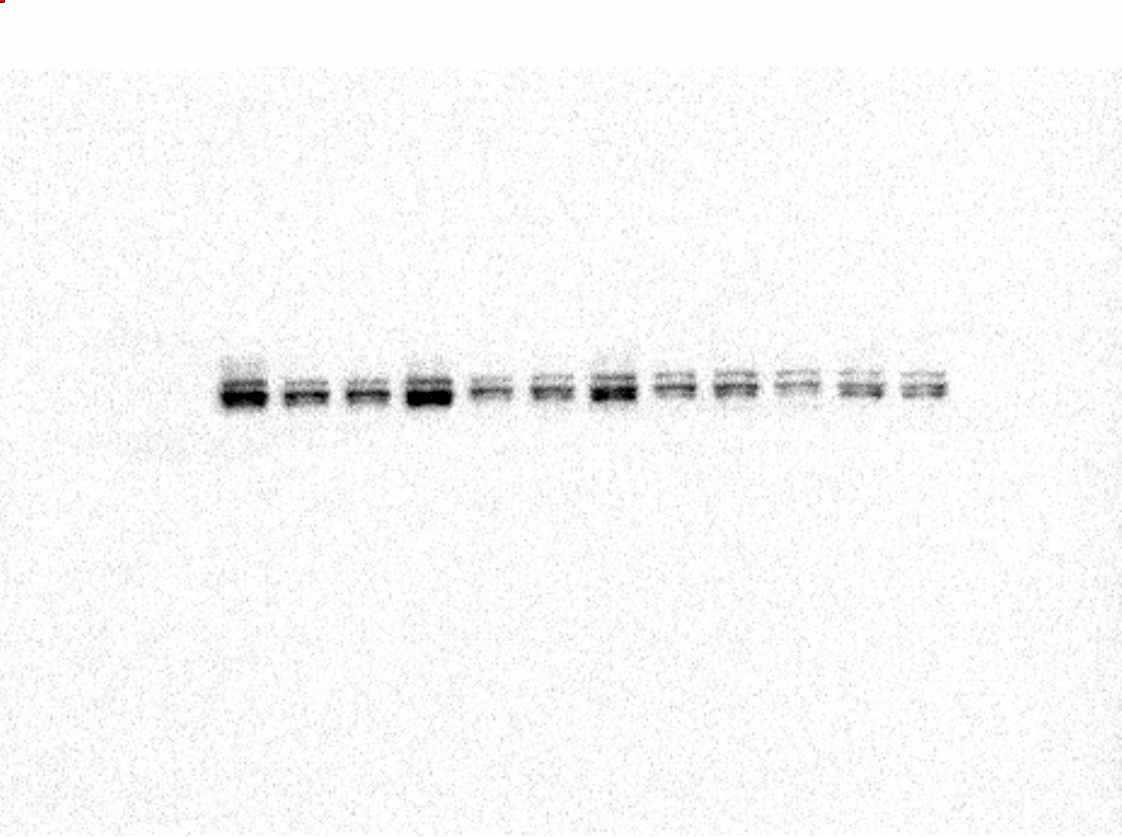

Supplement: Supplementary file 1 [file DataSheet1.zip › WB Images/NRG1(without intervention)/20230307-105.tif]

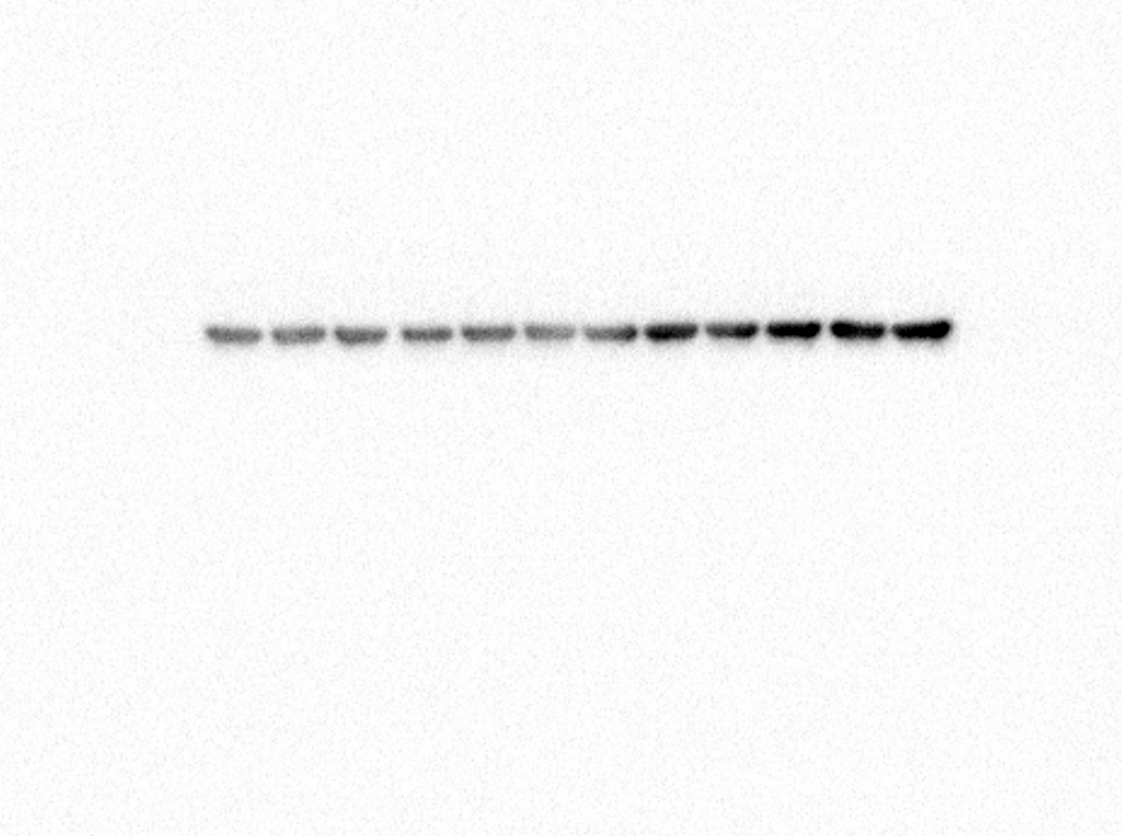

Supplement: Supplementary file 1 [file DataSheet1.zip › WB Images/β-actin(intervened)/2023-10-11 (CRS)CRSCRR(CRR).tif]

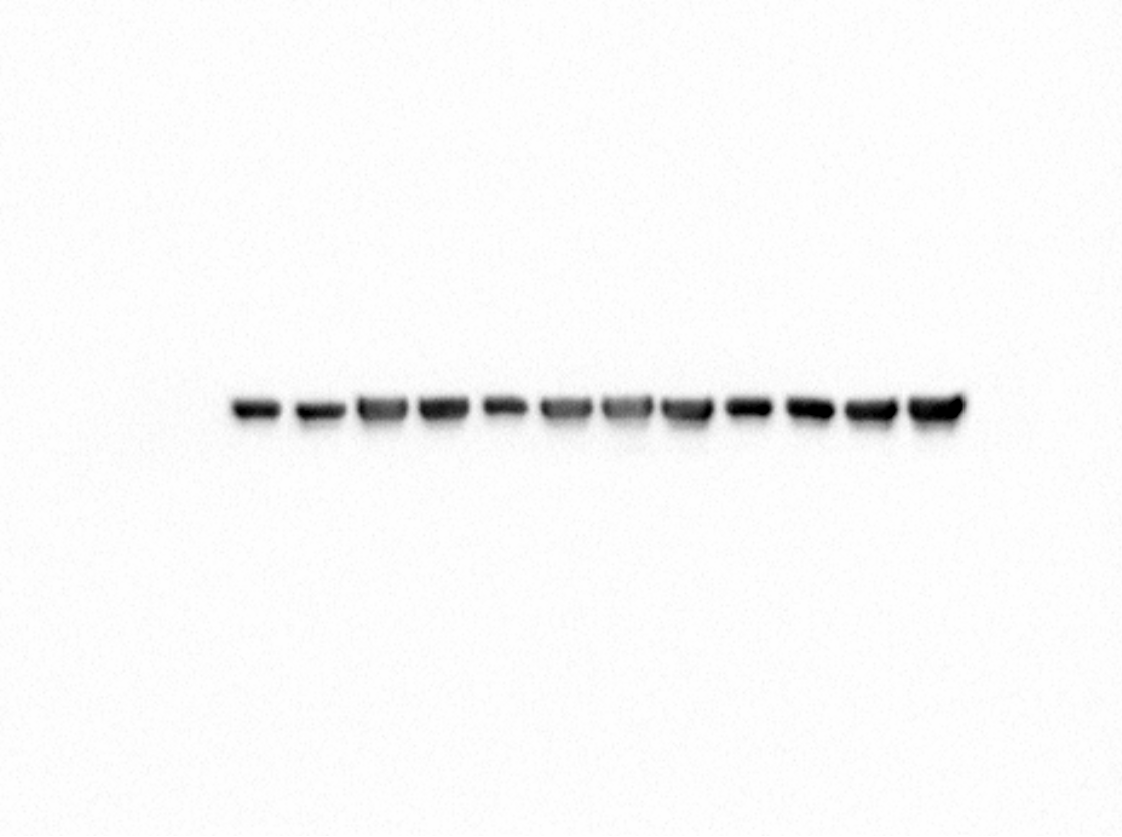

Supplement: Supplementary file 1 [file DataSheet1.zip › WB Images/β-actin(intervened)/2023-10-19 (CRS)(CRS)CRSCRS.tif]

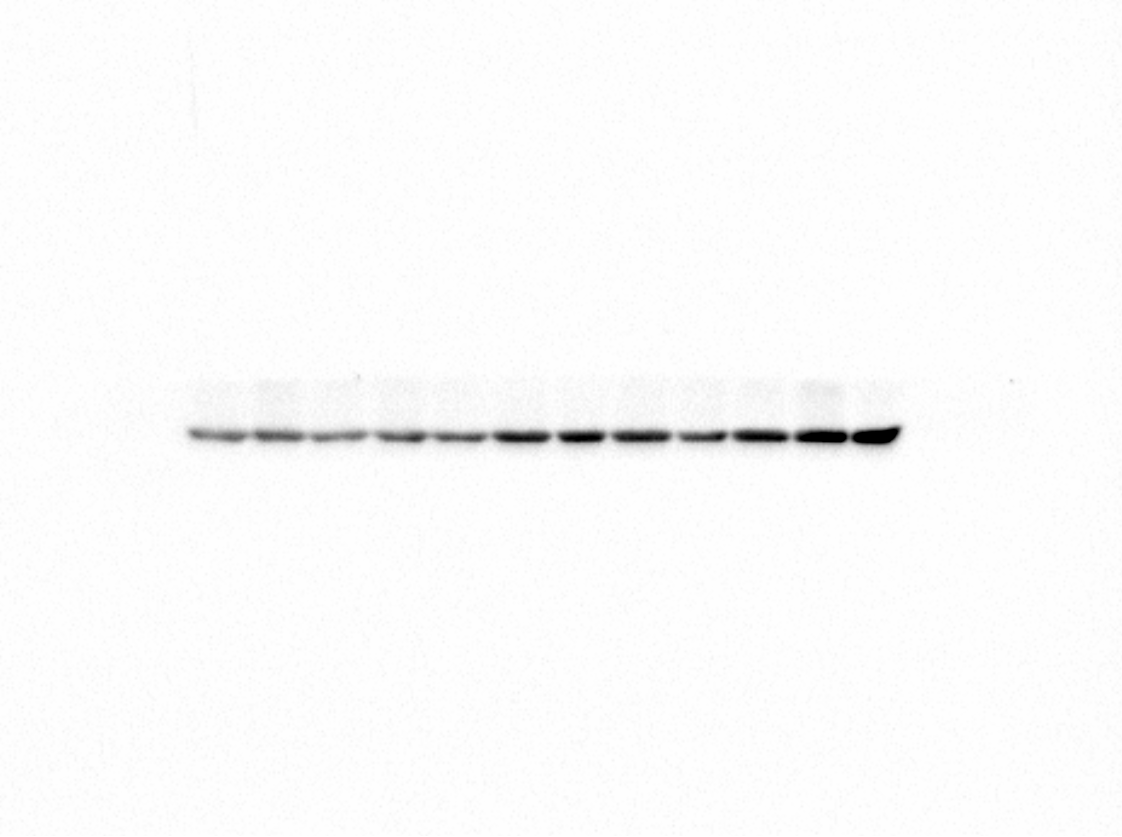

Supplement: Supplementary file 1 [file DataSheet1.zip › WB Images/β-actin(intervened)/2024-03-26CRS(CRSCRS)CRS .tif]

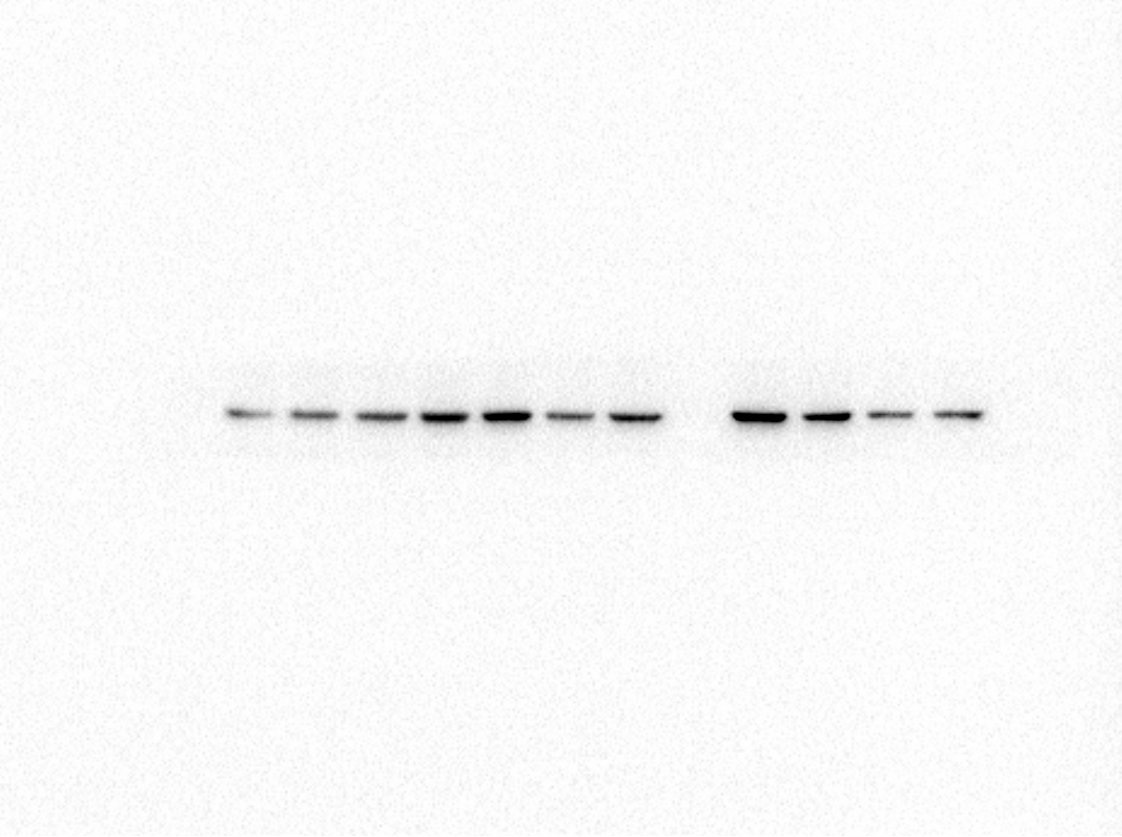

Supplement: Supplementary file 1 [file DataSheet1.zip › WB Images/β-actin(intervened)/43Administrator 2023-05-24 11 时 34 分_Exposure_53.8sec.tif]

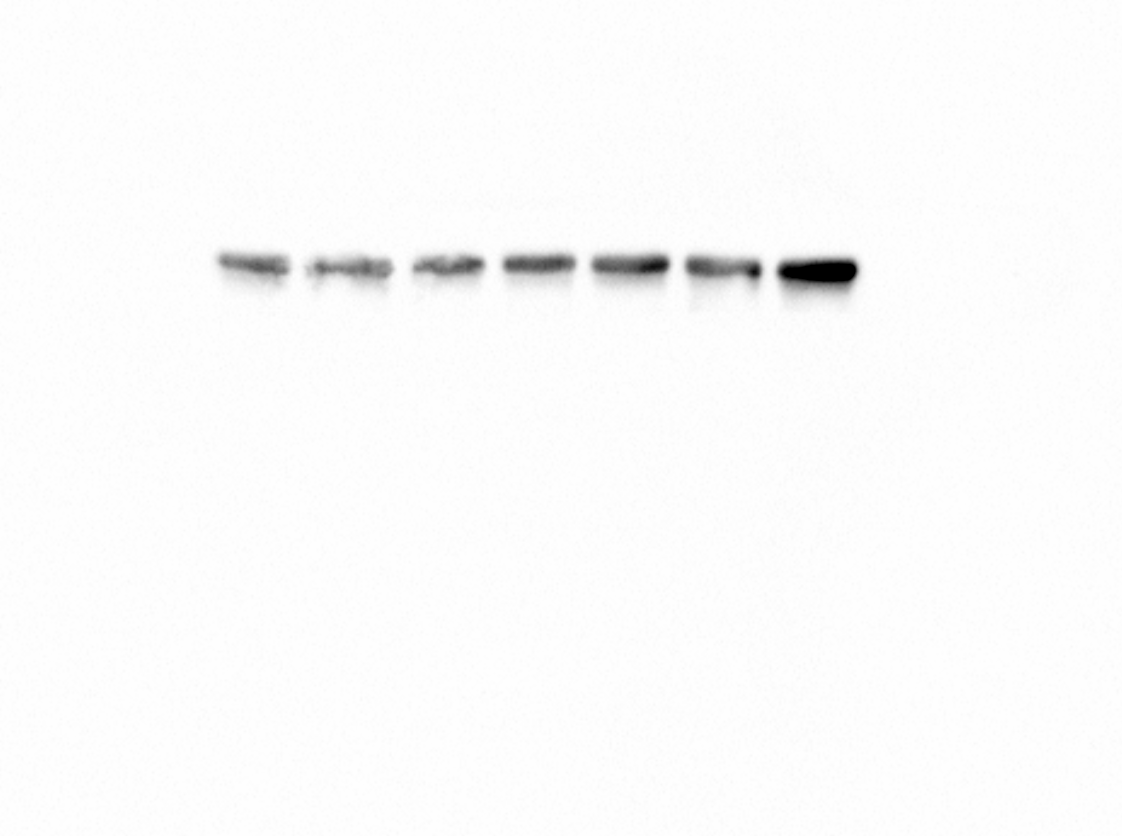

Supplement: Supplementary file 1 [file DataSheet1.zip › WB Images/β-actin(without intervention)/20221126-43.tif]

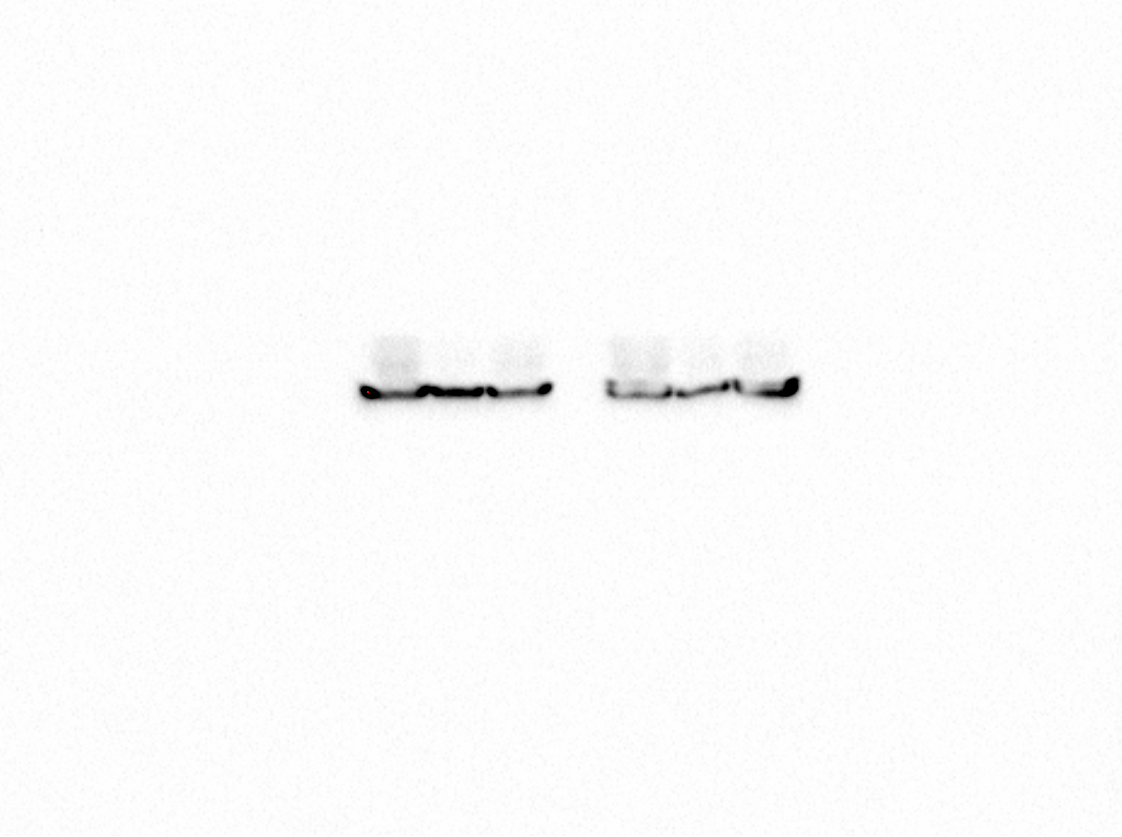

Supplement: Supplementary file 1 [file DataSheet1.zip › WB Images/β-actin(without intervention)/20230303-六个-43.tif]

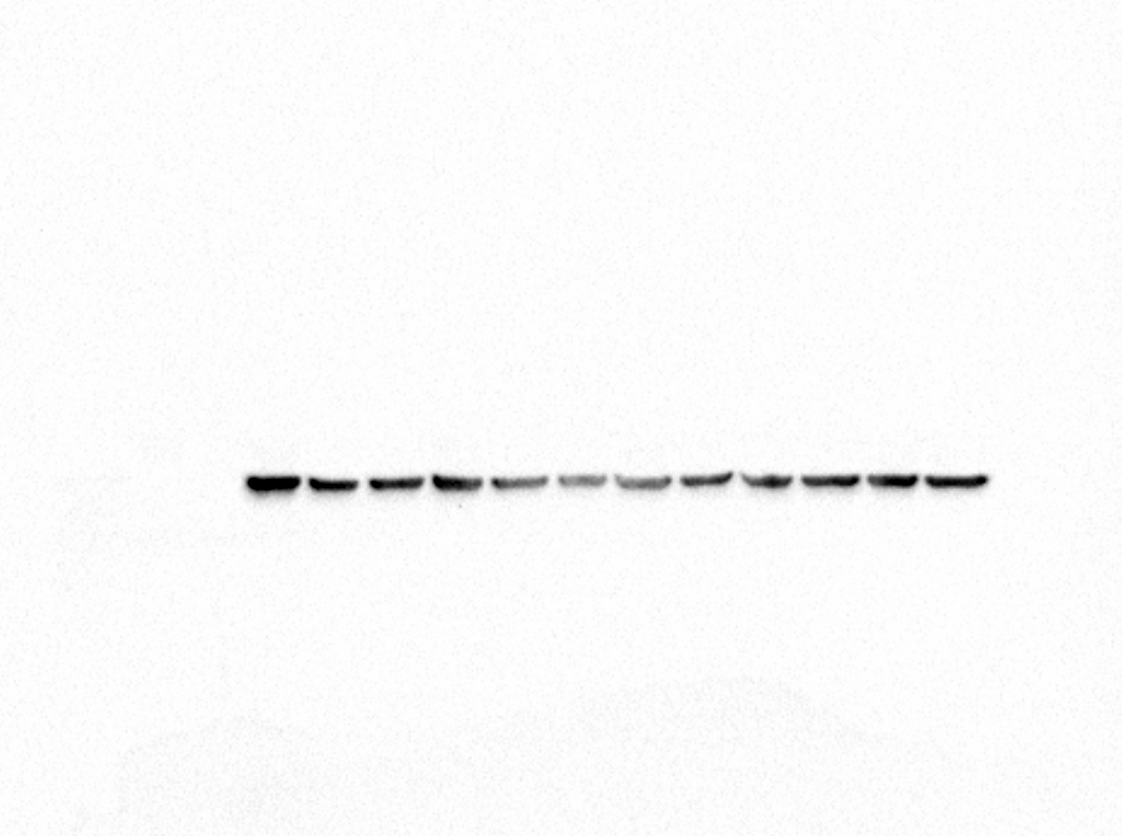

Supplement: Supplementary file 1 [file DataSheet1.zip › WB Images/β-actin(without intervention)/20230307-43.tif]
